# Combined Subchronic Toxicity of Aluminum (III), Titanium (IV) and Silicon (IV) Oxide Nanoparticles and Its Alleviation with a Complex of Bioprotectors

**DOI:** 10.3390/ijms19030837

**Published:** 2018-03-13

**Authors:** Ilzira A. Minigalieva, Boris A. Katsnelson, Larisa I. Privalova, Marina P. Sutunkova, Vladimir B. Gurvich, Vladimir Y. Shur, Ekaterina V. Shishkina, Irene E. Valamina, Oleg H. Makeyev, Vladimir G. Panov, Anatoly N. Varaksin, Tatiana V. Bushueva, Renata R. Sakhautdinova, Svetlana V. Klinova, Svetlana N. Solovyeva, Ekaterina Y. Meshtcheryakova

**Affiliations:** 1 The Medical Research Center for Prophylaxis and Health Protection in Industrial Workers, 30 Popov Str., Ekaterinburg 620014, Russia; ilzira-minigalieva@yandex.ru (I.A.M.); privalovali@yahoo.com (L.I.P.); marinasutunkova@yandex.ru (M.P.S.); gurvich@ymrc.ru (V.B.G.); bushueva@ymrc.ru (T.V.B); sahautdinova@ymrc.ru (R.R.S); klinova@ymrc.ru (S.V.K.); solovyeva@ymrc.ru (S.N.S.); 2 The Institute of Natural Sciences, The Ural Federal University, Ekaterinburg 620000, Russia; vladimir.shur@urfu.ru (V.Y.S.); ekaterina.shishkina@labfer.usu.ru (E.V.S.); 3 The Central Research Laboratory, The Ural State Medical University, 17 Klyuchevskaya Str., Ekaterinburg 620109, Russia; ivalamina@mail.ru (I.E.V.); ommt305@mail.ru (O.H.M.); katusha-ugma@rambler.ru (E.Y.M.); 4 Institute of Industrial Ecology, the Urals Branch of the Russian Academy of Sciences, 20 Sofia Kovalevskaya Str., Ekaterinburg 620990, Russia; vpanov@ecko.uran.ru (V.G.P.); varaksin@ecko.uran.ru (A.N.V.)

**Keywords:** nanoparticles, subchronic effects, comparative and combined toxicity, bioprotectors

## Abstract

Stable suspensions of metal/metalloid oxide nanoparticles (MeO-NPs) obtained by laser ablation of 99.99% pure elemental aluminum, titanium or silicon under a layer of deionized water were used separately, or in three binary combinations, or in a ternary combination to induce subchronic intoxications in rats. To this end, the MeO-NPs were repeatedly injected intraperitoneally (i.p.) 18 times during 6 weeks before measuring a large number of functional, biochemical, morphological and cytological indices for the organism’s status. In many respects, the Al_2_O_3_-NP was found to be the most toxic species alone and the most dangerous component of the combinations studied. Mathematical modeling with the help of the Response Surface Methodology showed that, as well as in the case of any other binary toxic combinations previously investigated by us, the organism’s response to a simultaneous exposure to any two of the MeO-NP species under study was characterized by a complex interaction between all possible types of combined toxicity (additivity, subadditivity or superadditivity of unidirectional action and different variants of opposite effects) depending on which outcome this type was estimated for and on effect and dose levels. With any third MeO-NP species acting in the background, the type of combined toxicity displayed by the other two remained virtually the same or changed significantly, becoming either more or less unfavorable. Various harmful effects produced by the (Al_2_O_3_-NP + TiO_2_-NP + SiO_2_-NP)-combination, including its genotoxicity, were substantially attenuated by giving the rats per os during the entire exposure period a complex of innocuous bioactive substances expected to increase the organism’s antitoxic resistance.

## 1. Introduction

The toxicity of metal nanoparticles and, especially, of metal oxide ones (MeO-NPs) has been a subject of extensive studies conducted by our team for the last few years [1,2,3,4,5,6,7,8,9,10,11,12,13,14,15,16,17,18]. Apart from a range of purposely manufactured (“engineered”) NPs, very important are their “natural” analogues always present in the workplace and ambient air of arc-welding and metallurgical operations. Such MeO-NPs constitute a substantial fraction in the particle size distribution of the polluting condensation aerosols (see examples in [17]). Meanwhile, it is multiple-factor rather than single-agent potentially hazardous nano-impacts on human health that are a common feature of these environments. Thus, the MeO-NP mixture generated by arc-welding and alloyed steel metallurgy usually comprises oxides of iron, manganese, nickel, chrome, vanadium, silicon and other elements. In nonferrous metallurgies, the typical factors are combined exposures to some of the just listed or to some other MeO-NPs (e.g., PbO, CuO, and ZnO in copper smelting and refining).

Both the chemical identity of these NPs and quantitative relationships between them vary broadly depending on a specific technology or its phase, on the composition of the alloy being molten or welded and welding electrodes being used, on the melting temperature, etc. As well as seeking to identify typical patterns and develop further the general theory of combined nano-metal toxicity, our studies therefore aimed to provide specific estimates of it with reference to some actual industrial exposure settings. This explains the choice of the MeO-NP combinations considered in this paper.

Samples of airborne micro- and nano-particles were collected on polycarbonate filters at an aluminum-titanium alloy production facility. The elemental composition of the samples was determined by energy-dispersive analysis with the help of an electron-scanning microscope, AURIGA CrossBeam (Carl Zeiss, Oberkochen, Germany). As follows from the averaged data of Table 1, the largest shares belong to three chemical elements: titanium (17.5%), aluminum (14.8%) and silicon (12.0%), which together account for nearly half of the 18 identified elements (disregarding carbon and oxygen since they were components of the filter itself).

Based on these data, we chose TiO_2_-NP, SiO_2_-NP and Al_2_O_3_-NP for experimental assessment of their individual and combined toxicity.

The first two of the above species belong to the most widely manufactured and used nanomaterials [19] This explains why they are frequently subjected to toxicological assessment, though in vitro as a rule, and much less often in short-term animal experiments. The scientific literature of this kind dealing with TiO_2_-NP toxicity may be exemplified by [20,21,22,23,24,25,26,27,28,29,30,31,32], and that on SiO_2_-NP toxicity by [33,34,35,36,37,38,39,40,41,42,43,44,45,46,47] and numerous other sources. Apart from several studies performed on unicellular algae or plant cells, the toxicity of Al_2_O_3_-NPs had been investigated much less, even on cell cultures [48,49,50], and only one of the latter [50] also involved the administration of a single oral dose to mice. We have failed to find any publication devoted to the comparative and combined toxicity of the three MeO-NP species under consideration or even of any pair of them.

At the same time, it would be of practical importance to find bioprotectors which, if administered in innocuous doses, could enhance the resistance of the organism to the effect of the MeO-NP [11,17,51,52]. Interestingly, one of the very few studies on nano-toxicity attenuation performed outside our team was concerned with the hepatotoxicity of just TiO_2_-NP ameliorated by means of *Cinnamomum cassia* [28].

## 2. Results and Discussion

### 2.1. Functional and Biochemical Outcomes of Intoxication

All the measured experimental values related to functional and biochemical indices for the organism’s status are given in Tables S1 and S2 in the Supplementary Materials while here, for readers’ convenience, we present in Table 2 and Table 3 only those indices which were statistically significant different from at least one group of rats.

Comparison of toxic effects requires the same dose of each MeO-NP and we decided to apply 0.5 mg/mL. However, the Al_2_O_3_ suspension turned out to be not stable at this concentration. Therefore, we used half the Al_2_O_3_ dose (0.25 mg/mL). Nevertheless, it is noteworthy that even this dose of Al_2_O_3_-NP caused virtually the same changes in the majority of the indices as those induced by TiO_2_-NP or SiO_2_-NP at a twice higher dose, which indirectly points to a greater toxicity of Al_2_O_3_-NP. Moreover, some of the indices—reduced hemoglobin content, reduced hematocrit, more acidic pH and higher protein, urea, uric acid and creatinine contents of the urine, and a reduced mass coefficient of both kidneys—demonstrated a statistically significant greater impact of Al_2_O_3_-NP than that of the other two MeO-NP species.

Comparing the group-average values of the indices obtained in a given binary exposure group with the corresponding values of two groups exposed to respective MeO-NPs separately provides a tentative estimate of the combined toxicity pattern. Thus, for instance, the group (Al_2_O_3_-NP + TiO_2_-NP) is statistically significantly different from the group Al_2_O_3_-NP in six indices and from the group TiO_2_-NP in four indices. Note, in particular, that Al_2_O_3_-NP in this combination eliminated the inhibiting effect of the TiO_2_-NP acting alone on the exploring behavior indices. Besides, this combination lacked the GGTP activity inhibition caused by each of these МеО-NP species separately. A similar antagonistic type of combined toxicity follows from comparison of combined vs. separate action on the mass of both kidneys or on daily diuresis. On the contrary, a statistically significant *enhancement* of the effect produced by Al_2_O_3_-NP in the combination with TiO_2_-NP may be deduced from their effects on the blood ceruloplasmin level. It is true, however, that for the majority of the tabulated toxicodynamic indices the inter-group differences under consideration were either absent at all or statistically insignificant. Still, it is noteworthy that the deterioration of the general energy metabolism assessed by a statistically significant reduction in the activity of succinate dehydrogenase in blood lymphocytes under separate exposure to either Al_2_O_3_-NP or TiO_2_-NP was not revealed under their combined impact, i.e., we deal with subadditivity (antagonism) of unidirectional action.

The same Table 2 suggests that a similar effect-dependent ambiguity of the tentative combined toxicity classification holds if we compare the actions of the two other combinations with corresponding single-factor impacts.

**Figure 1:**
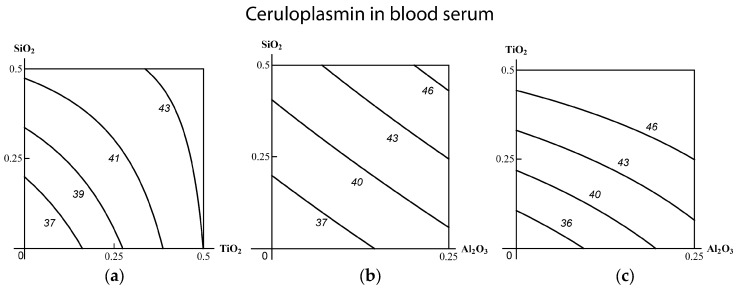
Combined subchronic toxicity isobolograms assessed by an increase in the concentration of ceruloplasmin in blood serum under exposure to (**a**) SiO_2_-NP + TiO_2_-NP (subadditivity); (**b**) SiO_2_-NP + Al_2_O_3_-NP (additivity); (**c**) TiO_2_-NP + Al_2_O_3_-NP (insignificant subadditivity). The axes represent doses of corresponding MeO-NPs in mg per rat; the numbers at the isoboles denote the magnitude of the effect (in mg per 100 mL). Note that the RSM-model (Response Surface Method) failed to reveal for this effect even the above-mentioned tendency towards superadditivity.

**Figure 2:**
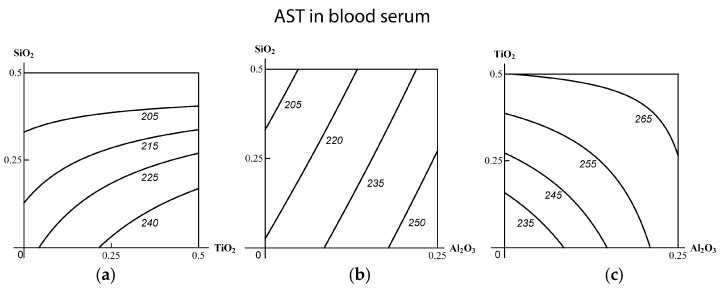
Combined subchronic toxicity isobolograms assessed by an increase in the concentration of AST in blood serum under exposure to (**а**) SiO_2_-NP + TiO_2_-NP (opposite action); (**b**) SiO_2_-NP + Al_2_O_3_-NP (opposite action); (**c**) TiO_2_-NP + Al_2_O_3_-NP (subadditivity of unidirectional action). The axes represent doses of corresponding MeO-NPs in mg per rat; the numbers at the isoboles denote the magnitude of the effect (in IU/L).

**Figure 3:**
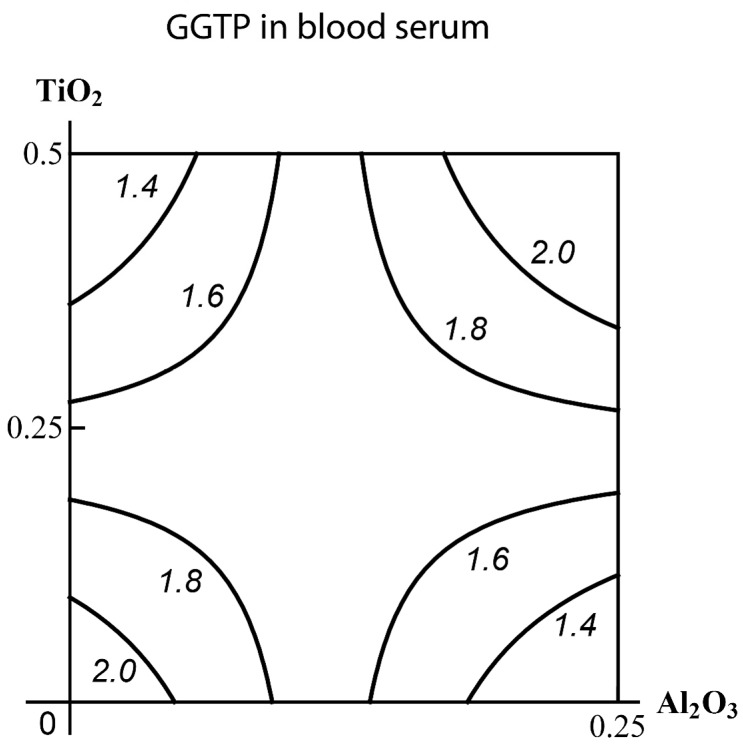
The ambiguity of the TiO_2_-NP + Al_2_O_3_-NP combined subchronic toxicity type assessed by a decrease in the GGTP content of blood serum: subadditivity of unidirectional action at low doses and relatively high levels of effect; superadditivity at high doses and similar levels of effect; opposite action at low doses and relatively low levels of effect and also at high doses and relatively low doses of effect. The axes represent doses of corresponding МеО-NPs in mg per rat; the numbers at the isoboles denote the magnitude of the effect (in IU/L).

We used mathematical modeling for confirming the effect-dependent and dose-dependent ambiguity of the combined binary action typology. Since this experiment is just an additional piece of evidence in support of this fundamental postulate, justified and repeatedly confirmed previously [12,15,17,53,54,55], we confine ourselves in this paper to illustrating it with a few examples only. Thus, comparison of Figure 1 and Figure 2 shows that the combination (SiO_2_-NP + TiO_2_-NP) displays subadditivity of unidirectional action for one effect (increase in the concentration of ceruloplasmin in the blood serum) and contra-directional action for another one (increase in AST concentration). Similarly, the combination (SiO_2_-NP + Al_2_O_3_-NP) demonstrates additive and opposite actions, respectively; and the combination (TiO_2_-NP + Al_2_O_3_-NP)—additivity and subadditivity of unidirectional action. An example of how the dependence of the type of combined toxicity varies for one and the same effect at different levels of it and different MeO-NP doses is illustrated by the isobologram in Figure 3.

As can be seen from Table 3, the toxic impact of the ternary combination assessed by shifts in the functional and biochemical indices of the organism’s status was not substantial. Moreover, there were almost no statistically significant distinctions from the same indices in the three groups of binary exposures.

Characterizing the type of combined toxic impact, which is ambiguous even for binary combinations, proves to be extremely complicated where three factors are involved. Previously, we had proposed [56] and then re-used [16] a two-phase risk-oriented analysis solving this problem.

In the first phase, we estimate all variants of combined toxicity for each of the three pairs of toxics involved in the ternary combination. In the second phase of analysis, all toxic exposure effects are classified depending on whether the type of combined toxicity displayed by one and the same pair is found, with a third factor added, to be more unfavorable for the organism (class А), less unfavorable for the organism (class В) or remains essentially unchanged in this respect (class С). Prior to carrying out such analyses, we defined criteria of such classification [56]. All previously conducted experiments with three-factor combinations of soluble salts or metal-oxide nanoparticles showed satisfactory stability of this classification. It was fully or partly reproducible when we considered as a third (background) factor one by one all components of a three-factor combination.

**Figure 4:**
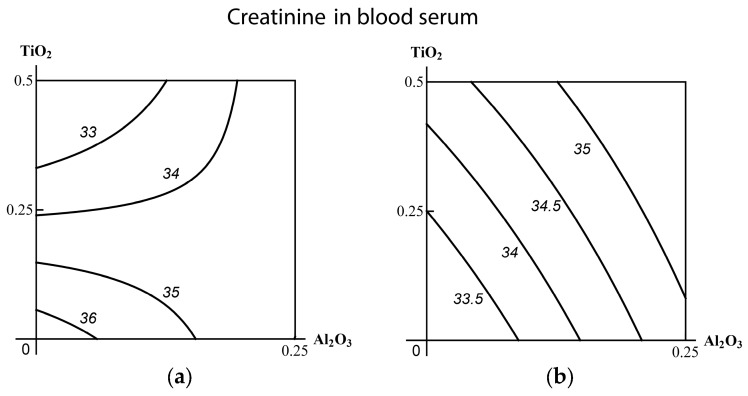
An example of three-factor toxicity falling into class “A”: (**а**) a subadditive or opposite action (for different levels of effect and doses) of the combination (Al_2_O_3_-NP + TiO_2_-NP) in the absence of a third factor on the creatinine content of blood serum transforms (for all effect levels and doses) into (**b**) an additive one in the presence of simultaneously influencing SiO_2_-NPs. Mе-NP doses are plotted on the axes in mg per rat. The numbers at the lines correspond to the magnitude of the effect (µmol/L).

Examples of effects falling into various classes based on the data of the current experiment are presented as isobolograms in Figure 4 (for the Class A) and in Figures S1 and S2 of the Supplementary Materials (for the Classes B and C, respectively).

On the whole, among all the effects of ternary toxic impacts classified reliably class А accounted for 35%, class В for 43%, and class С for 22%. Class А (42%) prevailed to some extent when Al_2_O_3_-NP was the third factor, while class В similarly prevailed (44%) in the case of the other two МеО-NP species.

### 2.2. Morphometry of the Most Characteristic Histological Changes in Kidneys and Liver 

**Figure 5:**
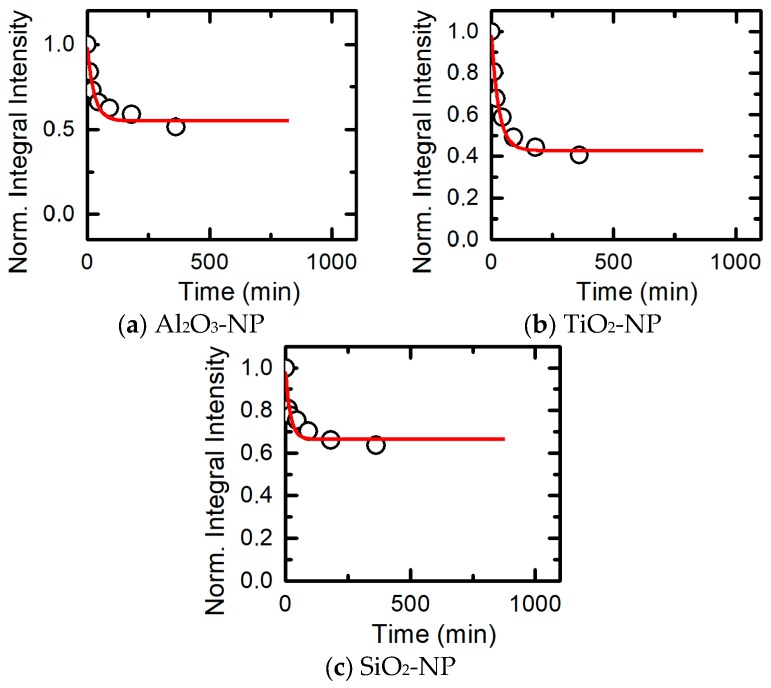
The kinetics of nanoparticle dissolution in suspension upon addition of FBS. Circles correspond to actual measurements at different time points during first 300 min, the red lines—to their approximation by exponential equations of the general formula: A + B exp(−t/τ) extrapolated to the 750 min.

As in all previously investigated subchronic intoxications with metal-containing nanoparticles, the most pronounced histopathological manifestation of renal toxicity was degenerative changes in the epithelium of the proximal convoluted tubules, including brush border loss, and, ultimately, complete epithelial desquamation. Table 4 shows that both adverse effects were most pronounced for the impact of TiO_2_-NP. Most likely, the metals impact directly on the kidneys not so much as persistent MeO-NP as in the form of ions released by them as a result of solubilization in biological milieus. We may therefore assume that the special nephrotoxicity of TiO_2_-NP is explained just by its highest (compared with Al_2_O_3_-NP and SiO_2_-NP) in vivo solubility which we modeled by adding fetal bovine serum (FBS) in vitro to each of the nano-suspensions (Figure 5).

It is easy to note that both indices under exposure to a binary combination comprising Al_2_O_3_-NP are higher than the corresponding values under an individual exposure to the second component of the combination. We could take as a measure of nephrotoxic effect the difference between the values of the corresponding index in the exposed and control groups. For the index of brush border loss, this difference is equal to 0.36 for Al_2_O_3_-NP, 2.12 for TiO_2_-NP, and 0.75 for SiO_2_-NP. Thus, the expected value of this difference upon full summation of the effects in the combination (Al_2_O_3_-NP + TiO_2_-NP) should have been equal to 2.48, but in reality we obtained 4.96. A similar expected value for (Аl_2_O_3_-NP + SiO_2_-NP) combination should be equal to 1.11 while actually it was found equal to 2.74. Respective estimates for the (TiO_2_-NP + SiO_2_-NP) combination are 2.87 and 2.15. Whereas in the latter case the impression is of an additive or slightly subadditive action, in the former two it appears to be more of a superadditive one. In the combination (Al_2_O_3_-NP + TiO_2_-NP) a similar tentative calculation also points to superadditivity.

Table 5 reproduces the same morphometric nephrotoxicity indices for the binary combinations in comparison with the corresponding indices of the ternary one (for full and half doses of each of the МеО-NP species in its composition). We may also note that the addition of SiO_2_-NP to the most nephrotoxic combination (Al_2_O_3_-NP + TiO_2_-NP) strengthened the effect but insignificantly (possible subadditivity of action). On the contrary, the addition of Al_2_O_3_-NP to the combination (SiO_2_-NP + TiO_2_-NP) increased the brush border loss making it statistically significant.

The relationship of this effect with the impact of the combination under study is confirmed by its explicit dependence on the dose of the whole combination (Table 5). At the same time, by way of preempting Section 2.5, let us note that the background administration of the bioprotective complex appears to have reduced the nephrotoxic effect of the ternary combination to a much greater extent than the halving of the dose.

Let us turn back to the tentative calculations carried out above based on the data of Table 4. One can calculate that the expected gain in the brush border loss compared with the control value index due to the effect of the three factors is equal to 3.23. Under the actual combined impact of these factors, however, it was found to be equal to 5.70. This again points to a likely prevalence of superadditive action. Similar summation of the three individual exposure values for the second nephrotoxicity effect (% epithelial desquamation) provides an estimate of 0.87 while the actual combined exposure value is 1.04, which also suggests superaddivity.

**Figure 6:**
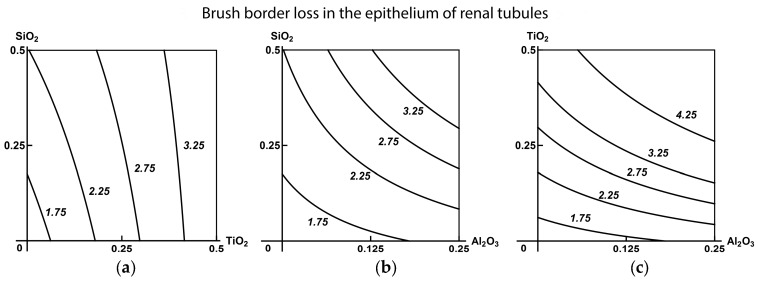
Isobolograms of combined subchronic toxicity assessed by brush border loss in the epithelium of renal tubules: (**а**) SiO_2_-NP + TiO_2_-NP (single-factor effect of TiO_2_-NP with insignificant additivity); (**b**) SiO_2_-NP + Al_2_O_3_-NP (additivity tending towards synergism); (**c**) TiO_2_-NP + Al_2_O_3_-NP (the same type of action). The axes represent doses of corresponding МеО-NPs in mg per rat. The numbers at the isoboles denote the magnitude of the effect (expressed in %—see the text).

Using RSM-modeling (Response Surface Method) again for predicting the type of combined nephrotoxicity outside the range of experimentally tested doses, we, on the whole, received support for the above tentative estimates. Indeed, as follows from the isobologram in Figure 6, only two binary combinations comprising Al_2_O_3_-NP revealed additive nephrotoxic action as judged by brush border loss with an insignificant, though clear departure from it towards synergism (superadditivity). On the contrary, the effect of the combination (SiO_2_-NP + TiO_2_-NP) is clearly dominated by the contribution of TiO_2_-NP, and it is only at minimal doses of the latter that we can see additivity or some subadditivity of the SiO_2_-NP action). Subadditivity of unidirectional action or even opposite action of this combination is clearly prevalent in the index of epithelial desquamation as well (see the Figure S3 in the Supplementary Materials).

**Figure 7:**
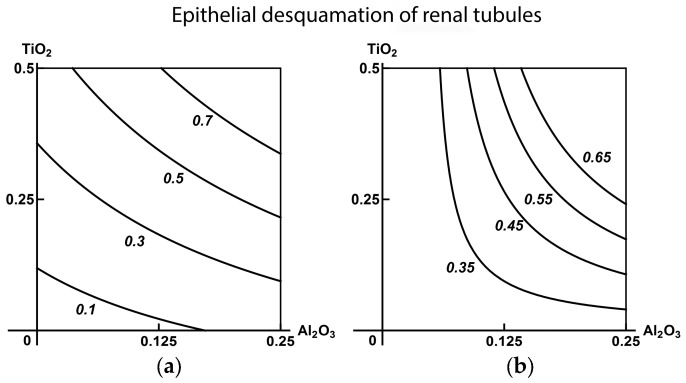
Isoboles of combined subchronic toxicity of Al_2_O_3_-NP + TiO_2_-NP assessed by epithelial desquamation of renal tubules: (**a**) additivity of unidirectional action in the absence of a third MeО-NP; (**b**) superadditivity in the presence of SiO_2_-NP (an example of a three-factor action falling within class А). The axes represent doses of corresponding МеО-NPs in mg per rat; the numbers at the isoboles denote the magnitude of the effect (expressed in %).

Again, the type of combined binary action may change more or less substantially under the influence of the third component of the combination as it is exemplified by Figure 7.

As well as in all our previous subchronic experiments with different MeO-NPs, the histological liver preparations displayed enhanced degeneration of the hepatocytes up to an increase in the number of cells which have lost their nuclei. As can be seen from Table 6 and Table 7, this quantitative index was increased in all exposure groups of this experiment too. Previously, we frequently saw also a decrease in the proportion of binuclear hepatocytes which may be interpreted as the evidence of reparative proliferation’s suppression. This effect was observed in the present case as well (Table 6 and Table 7). The number of Kupffer cells was, on the contrary, increased statistically significantly in all groups, though not that much. The latter shift could be associated either with the activation of this population of resident macrophages under the effect of nanoparticles engulfed by them or be an indirect sign of enhanced apoptosis of hepatocytes (considering the role of Kupffer cells in the utilization of apoptotic bodies [57]). Anyway, we had invariably observed it in our previous experiments with other МеО-NPs or Ме-NPs.

Comparison of the binary exposure groups with the groups exposed to corresponding two components individually (Table 6), and with the group exposed to the full ternary combination (Table 7) gives the impression of subadditivity as a predominant type of combined hepatotoxicity of the МеО-NPs under consideration. On the contrary, action on the spleen seems to be additive or even synergistic (Table 7).

**Figure 8:**
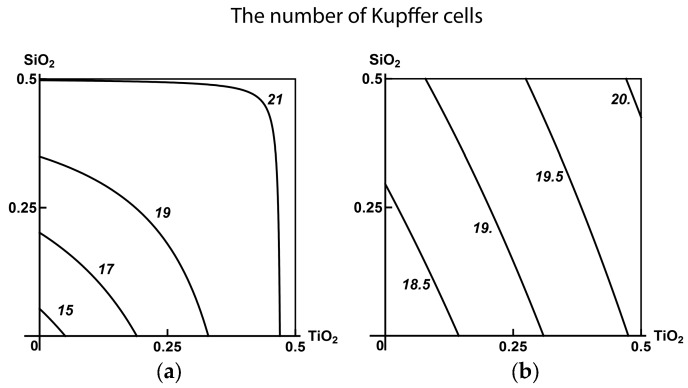
Isobolograms of combined subchronic toxicity of SiO_2_-NP + TiO_2_-NP assessed by an increase in the number of Kupffer cells: (**a**) subadditivity of unidirectional action in the absence of a third MeО-NP; (**b**) additivity in the presence of Al_2_O_3_-NP (an example of three-factor effect falling within class А). The axes represent doses of corresponding МеО-NPs in mg per rat; the numbers at the isoboles denote the magnitude of the effect (expressed in %—see the text).

These facts were again confirmed by RSM-modeling, which we illustrate with isobolograms in Figure 8 and Figure S6 (see Supplementary Materials). These charts, too, demonstrate that the introduction of a third component may change the type of combined action displayed by the other two, including towards greater adversity for the organism (class А). Note also that halving the dose of the ternary combination attenuated the hepatotoxic effect only in its direct morphometric indicator (i.e., the number of akaryotic hepatocytes).

### 2.3. Cytological Analysis of Tissue Imprints of Some Organs

In this experiment, we assessed damage to the cells of various organs in rats under subchronic nano-intoxication (single- and two-factor exposures) not only in situ on histological preparations but also by cytological analysis of tissue touch preparations (imprints).

Noteworthy is a certain essential similarity between the estimates obtained by this method and those commonly obtained in histological preparation morphometry. Indeed, the hepatotoxicity signs typically caused by the action of practically all МеO-NP species studied in our experiments were now found to be more or less substantial (with few exceptions) judging by the cytological data as well (Table 8). Of additional interest is the increased percentage of neutrophils and of eosinophils found under all MeO-NP exposures, which points to an inflammatory response of the hyperergic type. The dose of Al_2_О_3_-NP being half the dose of the other two MеО-NPs, the impression is that Al_2_О_3_-NP is the most hepatotoxic of all three species.

In principle, the same applies to the cytology of the kidney tissue imprints (Table 9). Note only that as well as in the histological examination, damage was observed predominantly in the cells of proximal (rather than distal) convoluted tubules, and the fact of toxic inflammation in this organ is evidenced by a largely eosinophilic reaction. In one case only, comparison of a binary action with a single-factor one seems to point to a statistically significant antagonism in the combination TiО_2_ + SiО_2_.

In the tissue imprints of both mesenteric lymph nodes (Table 10) and spleen (Table 11), conspicuous is a reduction in the percentage of lymphocytes with an increase in the percentage of other cell elements, mainly inflammatory cells. In the lymph node imprints, the total mature lymphocyte and prolymphocyte counts in the group (Al_2_О_3_ + SiО_2_) are significantly less than in the Al_2_О_3_ group and insignificantly greater than in the SiО_2_ group. This suggests subadditivity of toxic action, but in the other index (percentage of macrophages) that gave a statistically significant difference of the group (Al_2_О_3_ + SiО_2_) from each of the single-factor exposure groups, the sign of this difference is one and the same (additivity supposedly). The action of the combination (Al_2_О_3_ + ТiО_2_) also appears to be significantly additive according to the second index and subadditive according to the first one. In the spleen imprints, possible additivity of action in the combination (Al_2_О_3_ + SiО_2_) is suggested just by a unidirectional change in the percentage of lymphocytes.

Thus, even a descriptive analysis of the tissue imprint cytology data points again to a probable ambiguity of combined toxicity type for various organs and cells. This tentative statement was confirmed by RSM-based mathematical modeling. Typical results of this modeling are exemplified by several isoboles obtained for two combinations: Al_2_О_3_-NP+SiO_2_-NP (Figure S4) and Al_2_О_3_-NP + ТiO_2_-NP (Figure S5) (see the Supplementary Materials).

A similar typological variety of combined action judged by the cytological indices might also be demonstrated for the third binary combination (TiO_2_-NP + SiO_2_-NP). However, for the sake of reducing the length of this paper, we do not provide corresponding isoboles. Note only that we intentionally compare one and the same set of effects from two different binary combinations in order to illustrate the possibility of both mismatch and match between the types of combined toxicity in relation to a certain effect, even where these pairs differ in one factor only. Thus, for instance, the action of the pair (Al_2_О_3_-NP + SiO_2_-NP) on the percentage of degenerated hepatocytes proved to be opposite while that of the pair (Al_2_О_3_-NP + ТiO_2_-NP) was additive. At the same time, both combinations had an additive action on the percentage of degenerated tubular epithelium cells.

### 2.4. Genotoxic Effect

The genotoxic effect of the nanoparticles that we had studied previously in vivo was assessed by the “fragmentation coefficient” (C_fr_) in an RAPD assay on a genomic DNA isolated from cells of various organs and tissues. In a number of studies we have found that the genotoxic action of nanoparticles of silver and gold [7], manganese and nickel oxides [11], and copper, lead and zinc oxides [10,16] is *polyorganic*. Although the extent of increase in C_fr_ may be different for different organs (depending, supposedly, on the intensity of cell proliferation and on the degree of toxic damage to cells), the comparative genotoxicity of different NPs was usually independent of organ. To reduce effort and cost, we therefore thought it possible to confine ourselves to conducting RAPD testing on nucleated cells of circulating blood.

The results of this testing, performed using a similar experimental model of subchronic intoxication, are presented in Table 12. One can see that all three МеО-NPs provided a statistically significant genotoxic effect. That this effect is associated with the toxic exposure is evidenced by its dependence on the level of exposure (dose). Indeed, whereas the ternary combination of nanoparticles in full dose caused a 1.6 times increase in C_fr_ compared with the control value, the half dose of it brought about an increase of 1.2 times only (*p* < 0.05).

The genotoxic effect diminishes in the sequence Al_2_О_3_-NP >> TiО_2_-NP ≥ SiО_2_-NP, and in this respect, is similar to some integral, morphometric and cytological indices of the MeO-NPs systemic toxic impact discussed above. This rank agreement between systemic toxicity and genotoxicity is not surprising as the former is based on cytotoxicity, the widely presumed primary mechanisms of which include, in common with those of DNA damage, free radical generation and interactions of the nanoparticle surface and of metal ions released from it with membranes and bio-macromolecules. Note again that the higher genotoxic effect of Al_2_О_3_-NP compared with SiO_2_-NP and TiO_2_-NP appears to be especially convincing given the fact that the dose of the former was one half as high.

As follows from the same Table 12, the genotoxic effect of all three binary combinations is, to a degree, higher (though not always statistically significantly) than that of the МеО-NP species acting alone. Again, the presence of Al_2_О_3_-NP in the combination is of the greatest importance for this effect as well.

If we consider again the difference between the values of C_fr_ in each of the exposed and control groups as a measure of genotoxic effect, this effect appears to be superadditive in the Al_2_О_3_-NP-containing binary combinations (especially with TiО_2_-NP), as well as in the full ternary combination. In the binary combination (TiО_2_-NP + SiО_2_-NP), however, it looks as more of a subadditive type. To ascertain what type of combined action we were dealing with, we again resorted to RSM-modeling.

**Figure 9:**
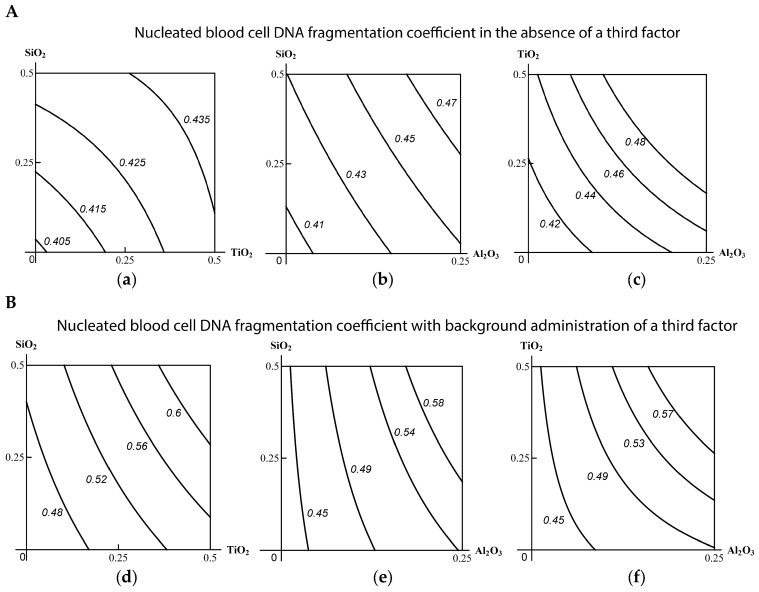
Isoboles of binary combined genotoxicity assessed by an increase in nucleated blood cell DNA fragmentation coefficient. (**А**) in the absence of a third factor under exposure to: (**а**) SiO_2_-NP + TiO_2_-NP (subadditivity); (**b**) SiO_2_-NP + Al_2_O_3_-NP (additivity); (**c**) TiO_2_-NP + Al_2_O_3_-NP (superadditivity). (**B**) with background administration of a third component: (**d**) Al_2_O_3_-NP to SiO_2_-NP + TiO_2_-NP (additivity); (**e**) TiO_2_-NP to SiO_2_-NP + Al_2_O_3_-NP (single-factor action with transformation into additivity); (**f**) SiO_2_-NP to ТiO_2_-NP + Al_2_O_3_-NP (superadditivity). The axes represent corresponding МеО-NPs in mg per rat; the numbers at the isobole denote the dimensionless quantity C_fr_.

As follows from the corresponding isoboles (Figure 9), there is subadditivity across the entire range of doses and responses for the pair (SiO_2_-NP + TiO_2_-NP). The pair (TiO_2_-NP + Al_2_O_3_-NP) displays undoubted superadditivity, which in the pair (SiO_2_-NP + Al_2_O_3_-NP) reveals itself as a slightly noticeable (and statistically insignificant) departure from additivity.

In the context of the above classification of three-factor combined toxicity, comparison of isoboles for this or that binary combination in the absence or presence of a third factor provides evidence of the following. When Al_2_O_3_-NP is considered to be such a background factor, the explicit subadditivity of the actions of the other two components transforms into an additivity with a tendency towards superadditivity. This type of combined action is more adverse for the organism (class А). If the third factor is TiO_2_-NP, the genotoxicity of the combination (SiO_2_-NP + Al_2_O_3_-NP), being strictly additive without this factor, approaches a single-factor one determined mainly by the dose of Al_2_O_3_-NP, which is less adverse (class В). Finally, the addition of SiO_2_-NP to the combination (ТiO_2_-NP + Al_2_O_3_-NP) did not actually change the superadditive type of its action (class С). However, bearing in mind the precaution principle, we believe it right to assess this ternary combination in whole as pertaining to the most adverse class А. This seems even more justified if we consider that this class was determined by the action of the factor (Al_2_O_3_-NP) that proved to be the most hazardous both alone and as a component of the binary combinations.

### 2.5. Efficacy of the Bioprotective Complex (BPC)

Turning back to the data presented in Table 3, we should note that the statistically significant protective effect of the BPC was established from the shifts caused by the ternary NP-combination in just a few indices (urea, reduced glutathione and ALT activity in blood serum, thrombocyte and reticulocyte counts). At the same time, a similar exposure with background administration of the BPC was accompanied by a statistically significantly enhanced leukocytosis, which, however, may be regarded as accidental because the action of the BPC by itself did not provoke this effect. BPC administration without exposure to the NPs gave a significant increase in the body mass compared with the controls. Therefore, the respective difference between the groups of ternary exposure without BPC and with BPC—being of the same sign but statistically insignificant—stands out as possibly beneficial. Moreover, in the second case the gain in body mass was higher than in the other groups which were not given the BPC, including the control one. The probability of such 5-fold coincidence being accidental is equal to 0.03 only.

**Figure 10:**
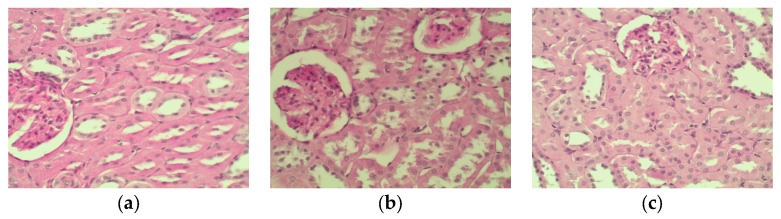
(**a**) Kidney of a control rat (proximal convoluted tubules with an intact brush border); (**b**) Kidney of a rat exposed to the ternary MeO-NP combination (marked degenerative and necrobiotic changes in tubular epithelial cells up to their disappearance; partial destruction of the brush border); (**c**) Kidney of a rat exposed to the same combination against background administration of the BPC. Periodic Acid Shiff (PAS) stain, magnification 400×.

A much more explicit protective efficacy of the BPC was shown by the morphometrically assessed indices of toxic damage to the internal organs, which in Figure 10 is exemplified by damage to kidneys.

As follows from Table 5, in this case the brush border loss index was equal for the three-factor intoxication to 7.19 ± 1.47, whereas for the same intoxication with background BPC administration to just 1.99 ± 0.43 (*p* < 0.05), only the former quantity being different from the control value (1.49 ± 0.56) statistically significantly. Inter-group differences of the same sign and statistical significance can be seen for epithelial desquamation as well. Note that the protective effect of the BPC proved more effective compared with that of twofold diminishing the toxic dose.

Table 7 demonstrates the protective effect of the BPC in all three morphometric indices of hepatotoxicity of the full ternary МеО-NP combination. However, it is statistically significant for one of them only (the most important one though), and in general is less pronounced than for the nephrotoxicity indices. The shift of the spleen red to white pulp ratio induced by the same МеО-NP combination was in the group administered the BPC two times and statistically significantly weaker than in one similarly exposed without protection.

Really striking results were obtained by RAPD assay (Section 2.4), which provided evidence of a high anti-genotoxic efficacy of the bioprotective complex tested. Indeed, as can been seen from Table 12, DNA fragmentation caused by exposure to the ternary MeO-NP combination was attenuated by the BPC even to greater degree than by halving the acting dose of the said combination. Meanwhile, attaining a twofold reduction in any harmful occupational exposure under actual industrial conditions presents a rather expensive and challenging task.

## 3. Materials and Methods

The experiment was carried out on outbred white male rats from our own breeding colony with the initial body weight of сa. 300 g, with a minimum of 12 animals in each of the exposed and control groups. The rats were housed in conventional conditions, breathed unfiltered air, and were fed standard balanced food. The experiments were planned and implemented in accordance with the “International guiding principles for biomedical research involving animals” developed by the Council for International Organizations of Medical Sciences (1985) and were approved on 20 January 2017 by the Ethics Committee of the Ekaterinburg Medical Research Center for Prophylaxis and Health Protection in Industrial Workers.

For this experiment, we prepared suspensions of metal oxide nanoparticles (MeO-NP) by laser ablation of 99.9% pure metal (Al and Ti) or semiconductor (Si) targets in sterile de-ionized water. The ablation was performed using an Fmark-20RL laser material processing system (Laser Technology Center, St. Petersburg, Russia) based on an ytterbium-doped pulsed fiber laser (pulse length 100 ns, repetition rate 21 kHz, wavelength 1064 nm). The energy density was about 80 J/cm^2^. The targets were irradiated in scanning mode with the rate of the laser spot at 270 mm/s. At the beginning, seven scanning cycles were used for preparation of the target surface.

A scanning electron microscope (SEM), CrossBeam Workstation Auriga (Carl Zeiss, Jena, Germany), was used for the visualization of the nanoparticles MPs. A Raman confocal microscope, Alpha 300 AR (WiTec, Ulm, Germany), was used for the analysis of the NP composition, found to be containing Al_2_O_3_, TiO_2_ and SiO_2_, respectively.

The concentration of the TiO_2_-NP and SiO_2_-NP suspensions was increased to 0.5 mg/mL by partial evaporation of the primary suspensions for 5 h at 50 °C. We were unable to concentrate the Al_2_O_3_-NP suspension without destabilization to any level greater than 0.25 mg/mL.

**Figure 11:**
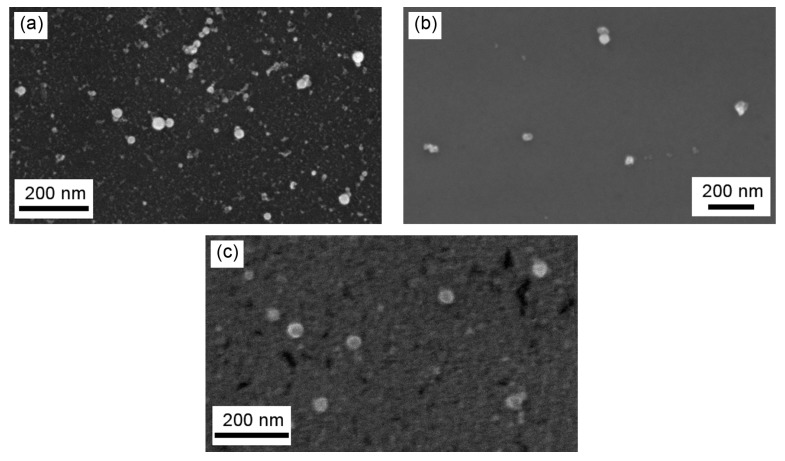
SEM images of (**a**) Al_2_O_3_; (**b**) TiO_2_ and (**c**) SiO_2_ nanoparticles in the suspensions prepared for this animal experiment.

**Figure 12:**
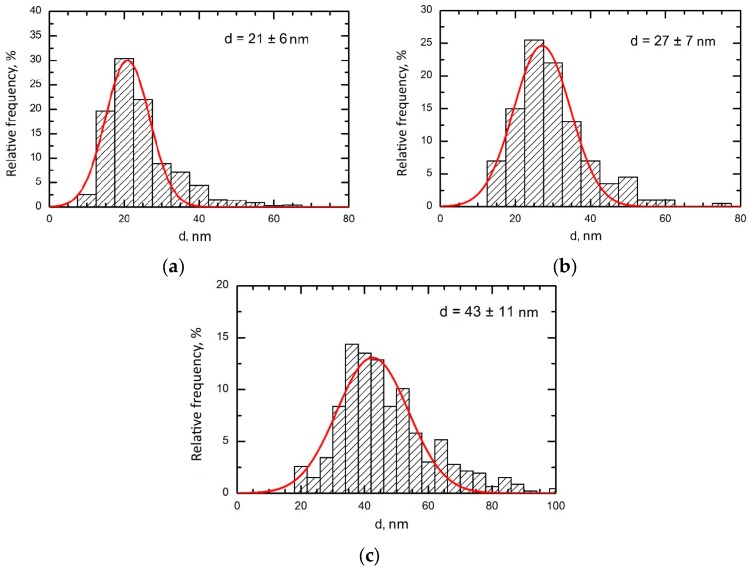
Size distribution functions obtained by statistical processing of SEM images of (**a**) Al_2_O_3_-NP; (**b**) TiO_2_-NP and (**c**) SiO_2_-NP. Arithmetic mean NP diameters (±s.d.) are shown within each panel.

The nanoparticles in all the suspensions were of spherical shape (Figure 11). The average particle diameter (±s.d.) obtained by statistical processing of hundreds of scanning electron microscopy (SEM) images was 21 ± 6 nm for Al_2_O_3_-NP, 27 ± 7 nm for TiO_2_-NP and 43 ± 11 for SiO_2_-NP. The distribution functions (Figure 12) were symmetrical in all three cases.

For the experimental modeling of subchronic intoxications, each MeO-NP species was administered to rats by intraperitoneal (IP) injections three times a week (up to 18 injections) at a dose of 0.5 mg of TiO_2_-NP and SiO_2_-NP or 0.25 mg of Al_2_O_3_-NP per rat (i.e., about 2.5 and 1.25 mg/kg body mass, respectively) in 1 mL of the suspension. To avoid in combined exposure groups direct interactions between chemically different MeO-NPs resulting in their fast aggregation, the suspensions were drawn into different syringes and injected separately, one after another, at an interval of about 1 min.

The groups of rats being investigated in parallel were administered either one МеО-NP species alone in the above-mentioned doses plus 2 mL of deionized water; or one of the three possible binary combinations of these МеО-NPs (Al_2_O_3_-NP + TiO_2_-NP; Al_2_O_3_-NP + TiO_2_-NP; SiO_2_-NP + TiO_2_-NP) plus 1 mL of deionized water; or a ternary combination of the same МеО-NP in the same doses; or the same ternary combination at half dose; or 3 mL of deionized water without any NPs. Thus, the total volume of intraperitoneally injected liquid was equal to 3 mL per rat in all groups. Half of the rats in the latter two of the above groups received throughout the exposure period a bioprotective complex (BPC), including:

(1) Glutamate as an effective cell membrane stabilizer acting through the intensification of ATP synthesis under exposure to the damaging effect of various cytotoxic particles and, at the same time, as one of the precursors of glutathione, which is a powerful cell protector against oxidative stress as, presumably, one of the key mechanisms underlying the cytotoxicity and genotoxicity of virtually all metallic NPs.
(2) The other two glutathione precursors: glycine and cysteine (the latter in a highly active and metabolically well available form of *N*-acetylcysteine).
(3) Other agents of the organism’s anti-oxidant system (vitamins А, Е, and С, and selenium).
(4) Omega-3 polyunsaturated fatty acids whose intracellular derivatives—eicosanoids—activate DNA replication and thus play an important part in its repair.
(5) Iodine, taking into consideration the well-known disturbances of the thyroid function caused by some metallic intoxications.
(6) Essential elements known to be antagonists of the metal that forms MeO-NPs under study.
(7) Pectin enterosorbent as an agent that hinders the re-absorption of toxic metals excreted into the intestines with bile.

The dosage formulations of these bioprotectors, their doses and mode of administration are given in Table 13.

We gave glutamate to the rats as 1.5% solution instead of drinking water ad libitum. The “Amber Dew” (Ecco-Plus Ltd.: Zhukovskiy, Russia), a fish oil preparation rich in PUFA mainly of the ω-3 group (24%), was administered through gavage at a dose of 1 mL per rat. Apple pectin enterosorbent (Promavtomatika Ltd.: Belgorod, Russia) was added to the rats’ food in a quantity corresponding to a dose of ca. 1000 mg/kg body weight. Commercial preparations of iodide, amino acids and vitamins available as tablets were crushed and added to another portion of the food in quantities corresponding to recommended daily intake of these micronutrients for rats (where such recommendations were known only for humans, a recalculation to the rats nutritional requirements was made based on the species standard metabolism ratio).

Taking into consideration that the standard balanced food presumably meets the normal nutritional requirements of a rat, we assumed that additional intake of the above-listed bioactive substances would meet the increased needs connected with the molecular mechanisms of metallic NP toxicity. Nevertheless, it had to be checked whether or not such presumed overloading with them would evoke any unfavorable effects. That is why in our experiment one group of rats was administered the same BPC but was not exposed to any toxicant.

Immediately after the end of the exposure period, the following procedures were performed for all rats:

- Weighing of body.
- Estimation of the CNS ability to evoke temporal summation of sub-threshold impulses (a variant of the withdrawal reflex and its facilitation by repeated electrical stimulations in an intact, conscious rat) [58].
- Recording of the number of head-dips into the holes of a hole-board (which is a simple but informative index of exploratory activity frequently used for studying the behavioral effects of toxicants and drugs) [59,60].
- Collection of daily urine for analysis of its output (diuresis), specific gravity (density), protein, total coproporphyrin, δ-aminolevulinic acid (δ-ALA), urea, uric acid, creatinine.

Then the rats were killed by quick decapitation and blood was collected by exsanguination. The liver, spleen, kidneys, and brain were weighed. The biochemical indices determined from the blood included reduced glutathione (GSH), total serum protein, albumin, globulin, bilirubin, ceruloplasmin, malonyl dialdehyde (MDA), alkaline phosphatase, alanine- and aspartate-transaminases (ALT, AST), catalase, gamma glutamyl transferase, SH-groups, urea, uric acid, creatinine, thyrotropic hormone of hypophysis, thyroxin, and triiodothyronine. For determining hemoglobin content, hematocrit, mean erythrocyte volume and for counting RBC, WBS and thrombocytes, we used the MYTHIC-18 auto-hematology analyzer (C2 Diagnostic. Montperllier, France). Reticulocyte percentage was counted on smears under optical microscopy after the supravital staining with brilliant cresyl blue. Cytochemical determination of succinate dehydrogenase (SDH) activity in lymphocytes was based on the reduction of nitrotetrazolium violet to formazan, the number of granules of which in a cell was counted under immersion microscopy.

All the clinical laboratory tests on blood and urine with the exception of the above stipulated ones were performed using well-known techniques described in many manuals [61].

Liver, spleen, kidney, and brain tissue sections were prepared from four rats in each treated and control group for histological examination by the hematoxilin and eosin stain and, when necessary, PAS, Nissl or Perl’s stain. For morphometric characterization of these tissues, we used the Avtandilov’s planimetric ocular grid and the image recognition programmed system CellSens (Olympus, Hamburg, Germany).

Tissue touching imprints were made from the surfaces of freshly cut liver, kidneys, spleen and mesenteric lymph nodes on a glass slide, which were dried at room temperature and stained by Leishman’s stain. The cell composition and signs of cell damage were estimated under a binocular light microscope, Carl Zeiss Primo Star with a USCMOS imaging camera at 100× and 1000×. Microscopy involved counting 100 cells from each lymph node imprint and 300 cells from the imprints of other organs.

The in vivo subchronic genotoxic effect was estimated with the Random Amplification of Polymorphic DNA (RAPD) test on blood nucleated cells. The samples were collected into special vessels cooled to −80 °C. These were then promptly delivered in cryo-containers to a specialized laboratory. To isolate DNA from the cells, a GenElute (Sigma, St. Louis, MO, USA) set of reagents was used in accordance with the manufacturer’s guidelines. The DNA content of the samples was determined spectrophotometrically (Ultraspec 1100 pro; Amersham Biosciences Ltd.: Amersham, UK); these were then frozen and stored at −84 °C in a kelvinator (Sanyo Electric Co., Ltd.: Moriguchi, Japan) till the beginning of the RAPD test. The method is based on the fact that, unlike a fragmented DNA, which, in the agarose gel electrophoresis, forms the so-called comet tail, a non-fragmented DNA has a very low degree of migration and stays virtually in the same place (comet head), the degree of migration being directly related to the degree of DNA fragmentation. DNA amplification was carried out using specific primers and tritiated nucleotides. To characterize the degree of damage to DNA we used the “coefficient of fragmentation”, i.e., the ratio of total radioactivity of all tail fractions to that of the head. Each blood sample was analyzed in three replications.

For all toxicity indices measured in this experiment, the statistical significance of the differences between the group arithmetic mean values was estimated using ANOVA test with Bonferroni correction for multiple comparisons, but the tables of results presented in this paper do not specify it for the groups, comparing which would be pointless.

Mathematical modeling of responses to binary exposures was based on the Response Surface Method (RSM) [62,63]. In this methodology, Equation (1) describing the response surface *Y* = *Y*(*x*_1_, *x*_2_) can be constructed by fitting its coefficients to experimental data.

*Y* = *f*(*x*_1_, *x*_2_) (1)

where *Y* is the quantitative effect (outcome) of a toxic exposure; *x*_1_ and *x*_2_ are the doses of the toxicants participating in the combination; *f*(*x*_1_, *x*_2_) is a regression equation with some numeric parameters. In the case of two-level exposures (even if one of the levels is equal to zero), the response surface may have one possible shape (hyperbolic paraboloid)

*Y* = *b*_0_ + *b*_1_*x*_1_ + *b*_2_*x*_2_ + *b*_12_*x*_1_*x*_2_ (2)

It is inferred that two agents produce a unidirectional effect on response *Y* if both one-way response functions *Y*(*x*_1_, 0) and *Y*(0, *x*_2_) either increase or decrease with an increase in *x*_1_ or *x*_2_; on the contrary, two agents are assumed to be acting contra-directionally (oppositely) if one function increases while the other decreases. This mathematical model enables one to predict the magnitude of response *Y* for any combination of toxicant doses within the experimental range for each of them (rather than at two factual points only). The sectioning of the response surface on different levels corresponding to different meanings of the outcome *Y* or of the doses *x*, provides a family of Loewe isoboles that may have the same or a different form and/or different slopes and thus render the interpretation of binary combined toxicity types both easy and illustrative. In Section 2, we therefore discussed the results of the RSM modeling presented just in this way.

For risk-oriented mathematical description of three-factorial toxicity, we took advantage of the original approach that we had proposed and used previously [16,56].

## 4. Conclusions

Compared with the manganese, nickel, lead, zinc and copper oxides nanoparticle that we had tested previously in experiments of similar design, the subchronic intoxications that developed under the impact of the МеО-NPs investigated in this study (Al_2_O_3_, TiO_2_, SiO_2_) were characterized by a relatively small number of functional and biochemical indices for changes in the organism’s integral status. We did not reveal any signs that would be specific to the toxicodynamics of this or that NP-forming metal tested. At the same time, the morphometric indices (in full agreement with what we had observed previously) and cytological indices of toxic damage to the kidneys, liver and spleen (measured for the first time in our studies) provided evidence that all the МеО-NP species studied are undoubtedly toxic for several target organs. This organ toxicity was found to be qualitatively of similar type for different NP species but pronounced to a different degree. Also in full agreement with the previously gained data, the RAPD test revealed that the nano-intoxications studied involved enhanced genomic DNA fragmentation. For the majority of these adverse effects, aluminum oxide nanoparticles proved to be the most noxious.

Concerning the typology of combined toxicity, our new results are also consistent with the previously obtained data not only for other МеО-NP species but also for metal ions. Our findings confirm that this typology may be ambiguous for one and the same pair of toxic agents depending on the dose ratio, on specific effect for which this toxicity is assessed and, often, on the level of this effect. Methodologically, a new confirmation has been obtained for the Response Surface Method as an adequate tool for mathematical modeling of combined toxicity.

The fact that one of the components in a ternary MeO-NP combination operating in the background may modify the type of combined toxicity displayed by the other two towards either a higher or lower risk or remain essentially unchanged was established by us first as a common pattern of three-factorial toxicity of metals in ion-molecular form [16]. Later on, it was confirmed in experiments with CuO, PbO-NP and ZnO nanoparticles [56] and again in the present study. Even though the most adverse variant of three-factorial toxicity has been shown only for a proportion of outcomes, we maintain that the precautionary principle should orient experts’ attention just to this variant when analyzing multi-factorial occupational health risks. At the same time, we have proved once more that even these additionally enhanced adverse health effects of a ternary nano-combination (including the additive or even superadditive genotoxicity) could be substantially attenuated with a complex of bioprotectors acting beneficially through various mechanisms. 

We maintain that in the light of the challenges of assessing and managing occupational health risks in a specific industry all the obtained results are of *practical value* as much as they are new for the *given* combination of toxics irrespective of the fact that they may be similar to those obtained for other combinations. At the same time, for the development of the *general theory* of combined nano-toxicity, it is just this fundamental repeatability of the most important inferences from the various studies of our team that we believe to be the most interesting and important finding.

## Figures and Tables

**Table 1:** Averaged Elemental Composition of the Aerosol Particles Collected on Filters from the Workspace Air of Aluminum-Titanium Alloy Production Facilities (expressed in % of Total Elements Less Carbon and Oxygen).

Element	Percentage Content
**Al**	**14.8**
As	0.1
Ca	8.2
Cl	5.6
Cr	2.5
Cu	0.1
F	1.1
Fe	2.9
K	3.8
Mg	11.4
Na	6.7
Pb	4.0
S	3.6
**Si**	**12.0**
Sn	0.5
**Ti**	**17.5**
Zn	5.2
Total	100

**Table 2:** Functional and Biochemical Indices of Rat Organism Status differing significantly from controls and/or those of other groups after 18 (during 6 Weeks) Intraperitoneal Injections of Suspensions of Various MeO-NP Species Administered Individually in Binary Combinations (x ± s.e.). The complete Table is given as Table S1 of Supporting Material; x, mean; s.e., standard error.

Index	Control	Al_2_O_3_	TiO_2_	SiO_2_	Al_2_O_3_ + TiO_2_	Al_2_O_3_ + SiO_2_	TiO_2_ + SiO_2_
		Group 1	Group 2	Group 3	Group 4	Group 5	Group 6
Number of head-dips into holes during 3 min	4.73 ± 0.94	2.64 ± 0.64 *	1.92 ± 0.36 *	2.82 ± 0.64	5.08 ± 1.02 ^2^	3.00 ± 0.59	4.42 ± 0.67 ^2^
Number of squares crossed during 3 min	8.18 ± 1.25	5.82 ± 1.09	5.67 ± 0.99	7.00 ± 0.86	7.58 ± 1.17	5.00 ± 0.55 *	7.58 ± 1.19
Left kidney mass, g/100 g body mass	0.30 ± 0.01	0.28 ± 0.01	0.30 ± 0.01 ^1^	0.30 ± 0.01 ^1^	0.30 ± 0.01 ^1^	0.29 ± 0.01	0.30 ± 0.01
Right kidney mass, g/100 g body mass	0.31 ± 0.01	0.28 ± 0.01 *	0.30 ± 0.01 ^1^	0.30 ± 0.01 ^1^	0.30 ± 0.01 ^1^	0.30 ± 0.01 ^1^	0.30 ± 0.01
Spleen mass, g/100 g body mass	0.17 ± 0.01	0.20 ± 0.01	0.17 ± 0.01 ^1^	0.19 ± 0.01	0.18 ± 0.01	0.17 ± 0.01 ^1,3^	0.18 ± 0.01
Right testicle mass, g/100 g body mass	0.55 ± 0.02	0.51 ± 0.01	0.52 ± 0.02	0.55 ± 0.01 ^1^	0.54 ± 0.01	0.55 ± 0.01 ^1^	0.53 ± 0.01
Brain mass, g/100 g body mass	0.62 ± 0.01	0.59 ± 0.01 *	0.58 ± 0.02 *	0.63 ± 0.01 ^1,2^	0.61 ± 0.01	0.61 ± 0.01	0.61 ± 0.01
Hemoglobin, g/L	158.89 ± 1.16	141.14 ± 1.99 *	149.00 ± 3.64 ^1,^ *	149.71 ± 2.74 ^1,^ *	147.33 ± 2.87 *	146.00 ± 1.51 *	151.25 ± 2.45 *
Erythrocytes, 10^12^ cells/L	7.93 ± 0.16	7.68 ± 0.32	7.23 ± 0.13 *	7.58 ± 0.14	7.61 ± 0.24	7.48 ± 0.11 *	7.52 ± 0.10 *
Average erythrocyte volume, µm^3^	54.69 ± 0.86	55.05 ± 0.87	54.29 ± 0.85	54.34 ± 0.51	52.17 ± 0.67 ^1,2,^ *	52.36 ± 0.57 ^1,3,^ *	54.94 ± 0.34
Reticulocytes, ‰	13.63 ± 1.65	25.64 ± 2.32 *	32.60 ± 3.01 *	26.63 ± 1.66 *	29.90 ± 1.28 *	25.86 ± 1.61 *	31.67 ± 2.73 *
Hematocrit, %	21.54 ± 0.21	19.73 ± 0.26 *	20.21 ± 0.52 *	20.60 ± 0.40 *	20.03 ± 0.69 *	19.59 ± 0.26 *	20.65 ± 0.28 *
Leukocytes, 10^3^/µL	7.20 ± 0.37	8.98 ± 0.86 *	8.40 ± 0.43 *	7.69 ± 0.67	9.10 ± 1.03	9.40 ± 0.83 *	7.85 ± 0.67
Banded neutrophils, %	1.50 ± 0.17	1.88 ± 0.30	0.88 ± 0.13 ^1^ *	1.88 ± 0.40 ^2^	1.00 ± 0.00 ^1,^ *	2.29 ± 0.29 *	1.38 ± 0.18 ^2^
Succinate dehydrogenase (SDH) in blood lymphocytes, number of formazan granules per 50 cells	589.45 ± 16.55	536.73 ± 17.43 *	539.36 ± 16.94 *	553.55 ± 17.46	562.67 ± 15.74	551.55 ± 20.54	530.42 ± 16.03 *
γ-glutamyl transpeptidase (GGTP), IU/L	2.26 ± 0.69	1.14 ± 0.38	1.14 ± 0.40	1.86 ± 0.69	2.48 ± 0.45 ^1,2^	3.98 ± 0.99 ^1^	0.74 ± 0.30
Glucose, mol/L	7.09 ± 0.26	6.80 ± 0.21	6.33 ± 0.25 *	6.80 ± 0.30	6.10 ± 0.30 *	6.64 ± 0.18	7.04 ± 0.29
Ceruloplasmin in blood serum, mg per 100 mL	33.14 ± 1.13	38.09 ± 1.56 *	42.03 ± 2.05 *	40.39 ± 1.50 *	44.06 ± 1.53 ^1,^ *	46.22 ± 2.35 ^1,3,^ *	42.88 ± 1.44 *
Malonyl dialdehyde (MDA) in blood serum, µmol/L	3.51 ± 0.49	3.99 ± 0.19	3.16 ± 0.28 ^1^	3.37 ± 0.31	3.56 ± 0.48	5.10 ± 0.37 ^1,3,^ *	4.57 ± 0.19 ^2,3,^ *
Catalase in blood serum, µmol/L	1.34 ± 0.25	1.30 ± 0.22	1.20 ± 0.27	1.12 ± 0.22	1.31 ± 0.22	1.18 ± 0.24	0.65 ± 0.12 *
Total protein content of blood serum, g/L	80.47 ± 1.42	76.81 ± 1.97	75.43 ± 1.40 *	75.36 ± 2.00 *	80.49 ± 2.01	78.20 ± 1.33	78.93 ± 2.15
Albumin content of blood serum, g/L	44.34 ± 0.61	39.49 ± 0.81 *	40.28 ± 1.35 *	40.25 ± 1.44 *	41.31 ± 1.05 *	39.58 ± 0.67 *	40.18 ± 1.24 *
Albumin/Globulin Ratio	1.24 ± 0.04	1.08 ± 0.06 *	1.17 ± 0.07	1.14 ± 0.06	1.06 ± 0.05 *	1.03 ± 0.03 *	1.05 ± 0.05 *
ALT activity in blood serum, IU/L	70.82 ± 3.24	72.70 ± 3.10	69.00 ± 4.19	58.55 ± 4.281 *	66.46 ± 4.41	66.50 ± 1.66	63.94 ± 3.32
Alkaline phosphatase, IU/L	193.64 ± 13.08	215.71 ± 14.74	216.61 ± 23.36	212.59 ± 26.36	222.55 ± 13.71	240.48 ± 21.89 *	236.53 ± 10.62 *
Creatinine in blood serum, µmol/L	36.33 ± 1.46	33.64 ± 1.09	30.80 ± 0.711 *	32.40 ± 1.29 *	34.46 ± 1.71	34.50 ± 1.49	33.89 ± 1.352
Concentration of Ca^2+^ in blood serum, mol/L	2.61 ± 0.03	2.53 ± 0.02 *	2.54 ± 0.05	2.55 ± 0.04	2.56 ± 0.04	2.57 ± 0.03	2.52 ± 0.05
Urea in blood serum, mmol/L	4.44 ± 0.34	3.69 ± 0.28	3.89 ± 0.45	3.29 ± 0.40 *	3.59 ± 0.36	3.71 ± 0.27	3.73 ± 0.37
Daily diuresis, mL	29.67 ± 4.36	21.17 ± 2.39	26.43 ± 3.88	28.43 ± 5.73	33.00 ± 2.50 ^1^	24.86 ± 2.20	31.71 ± 5.64
Creatinine in urine, mmol/L	1.57 ± 0.11	2.56 ± 0.27 *	2.15 ± 0.35	1.91 ± 0.161	1.54 ± 0.141	1.92 ± 0.131	1.85 ± 0.17
Protein in urine, g/L	190.43 ± 29.63	298.45 ± 32.35 *	216.55 ± 33.41	180.93 ± 17.09 ^1^	196.13 ± 20.43 ^1^	193.36 ± 20.67 ^1^	211.45 ± 37.18
Urine pH	7.17 ± 0.17	6.50 ± 0.26 *	7.33 ± 0.40	7.36 ± 0.30	6.79 ± 0.15	7.00 ± 0.29	6.93 ± 0.17
Urea in urine, mmol/L	229.30 ± 16.00	319.41 ± 29.85 *	278.06 ± 46.97	240.15 ± 25.77 ^1^	211.22 ± 17.47 ^1^	262.08 ± 19.23	238.15 ± 24.71

The asterisk * designates the values which are statistically significantly different from the respective control ones, and the superscript numbers those statistically significantly different from the corresponding groups denoted with a corresponding number (*p* < 0.05 by ANOVA test).

**Table 3:** Functional and Biochemical Indices of Rat Organism Status differing significantly from controls and/or those of other groups after 18 (during 6 Weeks) Intraperitoneal Injections of Suspensions of Various МеO-NP Species Administered in Binary or Ternary Combinations (x ± s.e.). The complete Table is given as Table S2 of Supporting Materials.

Index	Control	Al_2_O_3_ + TiO_2_	Al_2_O_3_ + SiO_2_	TiO_2_ + SiO_2_	Al_2_O_3_ + SiO_2_+ TiO_2_	Al_2_O_3_ + SiO_2_ + TiO_2_ and BPC	BPC
		Group 1	Group 2	Group 3	Group 4	Group 5	Group 6
Body mass gain, %	15.13 ± 1.85	13.41 ± 2.07	14.70 ± 1.86	12.58 ± 2.09	15.40 ± 2.02	18.34 ± 2.00	20.32 ± 1.41 *
Number of squares crossed during 3 min	8.18 ± 1.25	7.58 ± 1.17	5.00 ± 0.55 *	7.58 ± 1.19	6.50 ± 1.09	4.90 ± 1.14	7.78 ± 1.16
Brain mass, g/100 g body mass	0.62 ± 0.01	0.61 ± 0.01	0.61 ± 0.01	0.61 ± 0.01	0.59 ± 0.01 *	0.59 ± 0.01 *	0.61 ± 0.01
Hemoglobin, g/L	158.89 ± 1.16	147.33 ± 2.87 *	146.00 ± 1.51 *	151.25 ± 2.45 *	147.75 ± 2.28 *	143.26 ± 1.49 *	151.14 ± 1.92 *
Erythrocytes, 10^12^ cells/L	7.93 ± 0.16	7.61 ± 0.24	7.48 ± 0.11 *	7.52 ± 0.10 *	7.83 ± 0.17	7.75 ± 0.14	7.42 ± 0.10 *
Average erythrocyte volume, µm^3^	54.69 ± 0.86	52.17 ± 0.67 *	52.36 ± 0.57 *	54.94 ± 0.34	51.73 ± 0.93 *	51.88 ± 0.82 *	56.60 ± 0.61
Reticulocytes, ‰	13.63 ± 1.65	29.90 ± 1.28 *	25.86 ± 1.61 *	31.67 ± 2.73 *	no data	26.00 ± 0.88 *	17.67 ± 4.78
Hematocrit, %	21.54 ± 0.21	20.03 ± 0.69 *	19.59 ± 0.26 *	20.65 ± 0.28 *	20.20 ± 0.30 *	20.09 ± 0.45 *	21.00 ± 0.36
Thrombocytes,10^3^/µL	847.25 ± 25.41	831.75 ± 54.09	926.57 ± 27.89	880.50 ± 34.53	882.25 ± 36.87	979.56 ± 26.63 ^4,^ *	890.25 ± 36.39
Thrombocrit, %	0.23 ± 0.02	0.24 ± 0.02	0.27 ± 0.01	0.25 ± 0.01	0.26 ± 0.01	0.29 ± 0.01 ^4,^ *	0.27 ± 0.01
Leukocytes, 10^3^/µL	7.20 ± 0.37	9.10 ± 1.03	9.40 ± 0.83 *	7.85 ± 0.67	7.78 ± 0.66	10.04 ± 0.93 ^4,^ *	7.88 ± 0.46
Eosinophils %	2.20 ± 0.29	3.13 ± 0.48	3.57 ± 0.87	2.13 ± 0.40	3.00 ± 0.42	3.22 ± 0.36 *	3.13 ± 0.69
Banded neutrophils, %	1.50 ± 0.17	1.00 ± 0.00 *	2.29 ± 0.29 *	1.38 ± 0.18	1.63 ± 0.26	1.67 ± 0.33	1.25 ± 0.16
Succinate dehydrogenase (SDH) in blood lymphocytes, number of formazan granules per 50 cells	589.45 ± 16.55	562.67 ± 15.74	551.55 ± 20.54	530.42 ± 16.03 *	561.64 ± 15.99	559.50 ± 16.67	578.90 ± 14.48
Ceruloplasmin in blood serum, mg per 100 mL	33.14 ± 1.13	44.06 ± 1.53 *	46.22 ± 2.35 *	42.88 ± 1.44 *	42.61 ± 1.88 *	38.36 ± 2.71	30.54 ± 1.82
Malonyl dialdehyde (MDA) in blood serum, µmol/L	3.51 ± 0.49	3.56 ± 0.48	5.10 ± 0.37 *	4.57 ± 0.19 *	4.28 ± 0.29	4.20 ± 0.28	4.09 ± 0.19
Catalase in blood serum, µmol/L	1.34 ± 0.25	1.31 ± 0.22	1.18 ± 0.24	0.65 ± 0.12 *	1.10 ± 0.21	0.74 ± 0.25	0.86 ± 0.31
Reduced glutathione in whole blood, µmol/L	26.82 ± 1.19	26.20 ± 0.87	28.44 ± 1.473	26.00 ± 1.39	22.55 ± 1.41 *	26.39 ± 1.36 ^4^	28.17 ± 1.35
Albumin content of blood serum, g/L	44.34 ± 0.61	41.31 ± 1.05 *	39.58 ± 0.67 *	40.18 ± 1.24 *	41.91 ± 0.88 *	43.38 ± 0.94	44.91 ± 0.90
Albumin/Globulin Ratio	1.24 ± 0.04	1.06 ± 0.05 *	1.03 ± 0.03 *	1.05 ± 0.05 *	1.11 ± 0.04 *	1.17 ± 0.05	1.27 ± 0.05
ALT activity in blood serum, IU/L	70.82 ± 3.24	66.46 ± 4.41	66.50 ± 1.66	63.94 ± 3.32	66.75 ± 3.55	83.09 ± 5.13 ^4,^ *	84.98 ± 4.69 *
Alkaline phosphatase, IU/L	193.64 ± 13.08	222.55 ± 13.71	240.48 ± 21.89 *	236.53 ± 10.62 *	200.30 ± 12.15	209.78 ± 21.48	261.99 ± 24.46 *
Urea in blood serum, mmol/L	4.44 ± 0.34	3.59 ± 0.36	3.71 ± 0.27	3.73 ± 0.37	3.35 ± 0.42	4.93 ± 0.45 ^4^	4.49 ± 0.42
Creatinine in urine, mmol/L	1.57 ± 0.11	1.54 ± 0.14	1.92 ± 0.13	1.85 ± 0.17	2.03 ± 0.19 *	1.74 ± 0.25	1.43 ± 0.21
Protein in urine, g/L	190.43 ± 29.63	196.13 ± 20.43	193.36 ± 20.67	211.45 ± 37.18	233.13 ± 30.83	354.33 ± 66.07	243.93 ± 22.83 *

The asterisk * designates the values which are statistically significantly different from the control ones, and the superscript numbers mark the values which are statistically significantly different from the corresponding values of the groups denoted with a corresponding number (*p* < 0.05 by ANOVA test).

**Table 4:** Morphometric Indices of Damage to the Epithelium of the Proximal Convoluted Tubules in Rat Kidneys after Subchronic Exposure to Al_2_O_3_-NP, TiO_2_-NP and SiO_2_-NP Individually or in Binary Combinations (x ± s.e.).

Index	Group Exposed i.p. to МеО-NP						
	Control	Al_2_O_3_	TiO_2_	SiO_2_	Al_2_O_3_ + TiO_2_	Al_2_O_3_ + SiO_2_	TiO_2_ + SiO_2_
Brush border loss, %	1.49 ± 0.56	1.85 ± 0.47	3.61 ± 0.99 *	2.24 ± 0.58	6.45 ± 1.07 *^, x, @^	4.23 ± 0.80 *^, +, @^	3.64 ± 0.70 *
Epithelial desquamation, %	0.00 ± 0.00	0.15 ± 0.15	0.42 ± 0.36	0.30 ± 0.25	0.97 ± 0.48 *	0.29 ± 0.17	0.14 ± 0.14

Statistically significant (*p* < 0.05 by ANOVA test) difference: * from the control value, ^+^ from the value of the SiO_2_ group, ^х^ from the value of the ТiO_2_ group, ^@^ from the value of the Al_2_O_3_ group.

**Table 5:** Morphometric Indices of Damage to the Epithelium of the Proximal Convoluted Tubules in Rat Kidneys after Subchronic Exposure to Al_2_O_3_-NP, TiO_2_-NP and SiO_2_-NP in Binary or Ternary Combinations (x ± s.e.).

Index	Group Receiving i.p. МеО-NP						
	Control	Al_2_O_3_ + TiO_2_	Al_2_O_3_ + SiO_2_	TiO_2_ + SiO_2_	Al_2_O_3_ + TiO_2_ + SiO_2_ at Half Dose	Al_2_O_3_ + TiO_2_ + SiO_2_ in Full Doses	Al_2_O_3_ + TiO_2_ + SiO_2_ in Full Doses with BPC
Brush border loss, %	1.49 ± 0.56	6.45 ± 1.07 *	4.23 ± 0.80 *	3.64 ± 0.70 *^, #^	3.06 ± 0.84 ^#^	7.19 ± 1.47 *	1.99 ± 0.43 ^#^
Epithelial desquamation, %	0.00 ± 0.00	0.97 ± 0.48 *	0.29 ± 0.17	0.14 ± 0.14	0.66 ± 0.47	1.04 ± 0.39 *	0.18 ± 0.16 ^#^

Statistically significant (*p* < 0.05 by ANOVA test) difference * from control value, ^#^ from the value of the group administered the ternary combination in full dose without BPC.

**Table 6:** Morphometric Indices of Liver Status in Rats after Subchronic Exposure to Al_2_O_3_-NP, TiO_2_-NP and SiO_2_-NP Individually or in Binary Combinations (x ± s.e.).

Number of Cells of a Given Type per 100 Liver Cells	Group Receiving i.p. МеО-NP						
	Control	Al_2_O_3_	TiO_2_	SiO_2_	Al_2_O_3_ + TiO_2_	Al_2_O_3_ + SiO_2_	TiO_2_ + SiO_2_
Acaryotic hepatocytes	10.30 ± 1.09	17.60 ± 0.98 *	41.27 ± 1.36 *	39.67 ± 2.58 *	29.45 ± 1.47 *	41.88 ± 1.72 *	41.90 ± 1.48 *
Binucleated hepatocytes	6.65 ± 0.83	5.67 ± 0.55	4.13 ± 0.47 *	3.27 ± 0.46 *	5.13 ± 0.46	3.13 ± 0.37 *	3.75 ± 0.52 *
Kupffer cells	14.28 ± 0.45	18.07 ± 0.62 *	21.43 ± 0.68 *	21.03 ± 0.62 *	19.58 ± 0.60 *	18.80 ± 0.72 *	21.05 ± 0.53 *

The sign * denotes a statistically significant (*p* < 0.05 by ANOVA test) difference from the corresponding control value. Also, there is a statistically significant difference: (1) in all indices, between the group exposed to Al_2_O_3_-NP and the group of individual exposure to the other two МеО-NPs; (2) in the number of akaryotic hepatocytes, between the group exposed to (Al_2_O_3_-NP + TiO_2_-NP) and the groups of exposure to both components individually; between the group exposed to (Al_2_O_3_-NP + SiO_2_-NP) and the group of exposure to Al_2_O_3_-NP alone; between the group exposed to (Al_2_O_3_-NP + ТiO_2_-NP) and the group of exposure to Al_2_O_3_-NP alone; (3) in the number of binucleated hepatocytes, between the group exposed to (Al_2_O_3_-NP + SiO_2_-NP) and the group of exposure to Al_2_O_3_-NP alone; (4) in the number of Kupffer cells, between the group exposed to (Al_2_O_3_-NP + ТiO_2_-NP) and the group of exposure to TiO_2_-NP alone; between the group exposed to (Al_2_O_3_-NP + SiO_2_-NP) and the group of exposure to SiO_2_-NP alone.

**Table 7:** Morphometric Indices of Liver and Spleen Status in Rats after Subchronic Exposure to Al_2_O_3_-NP, TiO_2_-NP and SiO_2_-NP in Binary or Ternary Combination (x ± s.e.).

Morphometric Index	Group Receiving i.p. МеО-NP						
	Control	Al_2_O_3_ + TiO_2_	Al_2_O_3_ + SiO_2_	TiO_2_ + SiO_2_	Al_2_O_3_ + TiO_2_ + SiO_2_ at Half Dose	Al_2_O_3_ + TiO_2_ + SiO_2 B_ in Full Dose	Al_2_O_3_ + TiO_2_ + SiO_2_ in Full Dose with BPC
**Liver (per 100 cells)**							
Akaryotic hepatocytes	10.30 ± 1.09	29.45 ± 1.47 *	41.88 ± 1.72 *^, #^	41.90 ± 1.48 *^, #^	16.92 ± 0.81 *^, #^	31.85 ± 1.74 *	27.13 ± 1.20 *^, #^
Binucleated hepatocytes	6.65 ± 0.83	5.13 ± 0.46	3.13 ± 0.37 *	3.75 ± 0.52 *	5.00 ± 0.33 *	4.05 ± 0.78 *	3.35 ± 0.32 *
Kupffer cells	14.28 ± 0.45	19.58 ± 0.60 *	18.80 ± 0.72 *	21.05 ± 0.53 *	20.58 ± 0.48 *	20.08 ± 0.75 *	18.58 ± 0.53 *
**Spleen**							
White pulp to red pulp planimetric ratio	0.50 ± 0.03	0.62 ± 0.05 *^, #, @^	0.63 ± 0.04 *^, #, @^	0.75 ± 0.05 *^, #, @^	0.92 ± 0.06 *	0.95 ± 0.06 *	0.47 ± 0.04 ^#, @^

The sign * denotes a statistically significant (*p* < 0.05 by ANOVA test) difference from control value, ^#^ from the index of the group administered the ternary combination in full dose without BPC; ^@^ from the group administered the ternary combination at half dose without BPC.

**Table 8:** Some Cytological Characteristics of Rat Liver Tissue Imprints as a Percentage of Total Cell Count in Rats after Subchronic Exposure to Al_2_O_3_-NP, TiO_2_-NP and SiO_2_-NP Individually or in Binary Combinations (x ± s.e.).

Factor	Duct Epithelial Cells	Degenerated Hepatocytes	Binucleated Hepatocytes	Kupffer Cells	Neutrophils	Eosinophils
Al_2_О_3_-NP	10.51 ± 1.79	9.49 ± 1.71	0.68 ± 0.48	6.10 ± 1.39 *	10.17 ± 1.76 *	3.73 ± 1.10
TiО_2_-NP	9.52 ± 1.71 *	8.16 ± 1.60	0.68 ± 0.48	2.38 ± 0.89	8.50 ± 1.63 *	5.10 ± 1.28 *
SiО_2_-NP	14.29 ± 2.04	5.78 ± 1.36	1.36 ± 0.68	3.74 ± 1.11	8.16 ± 1.60 *	5.10 ± 1.28 *
Al_2_О_3_-NP + TiО_2_-NP	12.71 ± 1.95	11.34 ± 1.86 *	1.03 ± 0.59	4.81 ± 1.25	5.84 ± 1.37	2.06 ± 0.83
Al_2_О_3_ + SiО_2_-NP	11.90 ± 1.89	8.16 ± 1.60	1.02 ± 0.59	5.44 ± 1.32 *	6.80 ± 1.47	5.10 ± 1.28 *
TiО_2_ + SiО_2_-NP	11.41 ± 1.84	9.73 ± 1.72	0.67 ± 0.47	4.36 ± 1.18	9.73 ± 1.72 *	4.36 ± 1.18 *
Control	14.86 ± 2.07	6.42 ± 1.42	1.01 ± 0.58	2.03 ± 0.82	3.72 ± 1.10	1.35 ± 0.67

The asterisk * denotes values which are statistically significantly different from the control (*p* < 0.05 by ANOVA test).

**Table 9:** Some Cytological Characteristics of Kidney Tissue Imprints as a Percentage of Total Cell Count in Rats after Subchronic Exposure to Al_2_O_3_-NP, TiO_2_-NP and SiO_2_-NP Individually or in Binary Combinations (x ± s.e.).

Factor	Proximal Tubule Cells	Degenerated Cells of Proximal Tubules	Distal Tubule Cells	Degenerated Cells of Distal Tubules	Neutrophils	Eosinophils
Al_2_О_3_-NP	60.33 ± 2.82	13.00 ± 1.94	7.00 ± 1.47	8.33 ± 1.60	4.33 ± 1.18	2.33 ± 0.87
TiО_2_-NP	56.00 ± 2.87 *	14.00 ± 2.00	7.67 ± 1.54	8.33 ± 1.60	5.00 ± 1.26	5.00 ± 1.26 *
SiО_2_-NP	56.67 ± 2.86 *	14.67 ± 2.04	9.00 ± 1.65	6.00 ± 1.37	5.67 ± 1.33	2.00 ± 0.81
Al_2_О_3_-NP + TiО_2_-NP	58.67 ± 2.84 *	16.33 ± 2.13 *	8.00 ± 1.57	5.67 ± 1.33	4.00 ± 1.13	2.67 ± 0.93
Al_2_О_3_ + SiО_2_-NP	56.67 ± 2.86 *	16.00 ± 2.12 *	7.00 ± 1.47	9.00 ± 1.65	4.00 ± 1.13	2.67 ± 0.93
TiО_2_ + SiО_2_-NP	57.48 ± 2.85 *	14.29 ± 2.02	9.30 ± 1.67	7.64 ± 1.53	4.65 ± 1.21	1.66 ± 0.74 ^+^
Control	67.67 ± 2.70	10.00 ± 1.73	7.67 ± 1.54	5.00 ± 1.26	5.00 ± 1.26	0.67 ± 0.47

The asterisk * denotes values which are statistically significantly different from the control; the sign ^+^ denotes the difference from the group administered TiО_2_-NP alone (*p* < 0.05 by ANOVA test).

**Table 10:** Some Cytological Characteristics of Mesenteric Tissue Imprints as a Percentage of Total Cell Count in Rats after Subchronic Exposure to Al_2_O_3_-NP, TiO_2_-NP and SiO_2_-NP Individually or in Binary Combinations (x ± s.e.).

Factor	Mature Lymphocytes and Prolymphocytes	Lymphoblasts	Reticular Cells	Plasmocytes	Macrophages	Neutrophils	Eosinophils
Al_2_О_3_-NP	85.33 ± 2.04 *	2.67 ± 0.93	1.33 ± 0.66	3.33 ± 1.04	2.67 ± 0.93	1.33 ± 0.66	3.33 ± 1.04
TiО_2_-NP	85.33 ± 2.04 *	2.67 ± 0.93	1.67 ± 0.74	3.33 ± 1.04	4.00 ± 1.13	1.33 ± 0.66	1.67 ± 0.74
SiО_2_-NP	74.67 ± 2.51 *	3.00 ± 0.98	1.33 ± 0.66	9.00 ± 1.65 *	3.33 ± 1.04	0.33 ± 0.33	8.33 ± 1.60 *
Al_2_О_3_-NP + TiО_2_-NP	82.00 ± 2.72 *	1.50 ± 0.86	1.50 ± 0.86	6.00 ± 1.68 *	3.50 ± 1.30	1.50 ± 0.86	4.00 ± 1.39
Al_2_О_3_ + SiО_2_-NP	82.89 ± 2.18 *^, +^	1.34 ± 0.67	0.67 ± 0.47	3.02 ± 0.99 ^+^	7.05 ± 1.48 *^, @, +^	1.34 ± 0.67	3.69 ± 1.09 ^+^
TiО_2_ + SiО_2_-NP	81.40 ± 2.24 *^, +^	1.66 ± 0.74	1.33 ± 0.66	3.32 ± 1.03 ^+^	6.98 ± 1.47 *^, +^	1.99 ± 0.81	3.32 ± 1.03 ^+^
Control	90.67 ± 1.68	1.67 ± 0.74	1.00 ± 0.57	2.00 ± 0.81	1.67 ± 0.74	1.33 ± 0.66	1.67 ± 0.74

The asterisk * denotes values which are statistically significantly different from the control; the sign ^@^ denotes the difference from the group administered TiО_2_-NP alone; the sign ^+^ the difference from the group administered SiО_2_-NP alone (*p* < 0.05 by ANOVA test).

**Table 11:** Some Cytological Characteristics of Spleen Tissue Imprints as a Percentage of Total Cell Count in Rats after Subchronic Exposure to Al_2_O_3_-NP, TiO_2_-NP and SiO_2_-NP Individually or in Binary Combinations (x ± s.e.).

Factor	Lymphocytes	Lymphoblasts	Reticular Cells	Plasmocytes	Macrophages	Neutrophils	Eosinophils
Al_2_О_3_-NP	76.00 ± 2.47 *	1.00 ± 0.57	1.00 ± 0.57	3.00 ± 0.98	5.00 ± 1.26	7.00 ± 1.47 *	7.00 ± 1.47
TiО_2_-NP	75.08 ± 2.49 *	0.66 ± 0.47	0.66 ± 0.47	1.66 ± 0.74	4.65 ± 1.21	7.64 ± 1.53 *	9.63 ± 1.70 *
SiО_2_-NP	78.67 ± 2.37 *	0.33 ± 0.33	1.00 ± 0.57	1.00 ± 0.57	3.67 ± 1.09	5.67 ± 1.33	9.67 ± 1.71 *
Al_2_О_3_-NP + TiО_2_-NP	82.67 ± 2.19 ^+, @^	1.00 ± 0.57	0.67 ± 0.47	1.00 ± 0.57	3.67 ± 1.09	4.00 ± 1.13	7.00 ± 1.47
Al_2_О_3_ + SiО_2_-NP	79.33 ± 2.34 *	0.33 ± 0.33	0.67 ± 0.47	1.00 ± 0.57	4.67 ± 1.22	8.33 ± 1.60 *	5.67 ± 1.33
TiО_2_ + SiО_2_-NP	78.67 ± 2.37 *	1.00 ± 0.57	0.67 ± 0.47	1.33 ± 0.66	2.33 ± 0.87	8.33 ± 1.60 *	7.67 ± 1.54 *
Control	87.00 ± 1.94	0.67 ± 0.47	1.33 ± 0.66	1.67 ± 0.74	3.00 ± 0.98	2.67 ± 0.93	3.67 ± 1.09

The asterisk * denotes values which are statistically significantly different from the control; the sign ^@^ denotes the difference from the group administered TiО_2_-NP alone; the sign ^+^ the difference from the group administered Al_2_О_3_-NP alone (*p* < 0.05 by ANOVA test).

**Table 12:** Increase in the Coefficient of Genomic DNA Fragmentation (C_fr_) (as per RAPD Test) of Nucleated Blood Cells in Rats after 18 (during 6 Weeks) Repeated Intraperitoneal Injections of Suspensions of Various МеO-NPs Individually and in Binary or Ternary Combination (x ± s.e.).

MeO-NPs to Which Rats Were Exposed	C_fr_
Аl_2_О_3_-NP	0.4470 + 0.0038 *^, +, х^
TiО_2_-NP	0.4328 + 0.00548 *
SiО_2_-NP	0.4288 + 0.0061 *
Al_2_О_3_-NP + TiО_2_-NP	0.5416 + 0.0046 *^, @^
Al_2_О_3_-NP + SiО_2_-NP	0.4872 + 0.0041 *^, х, @^
TiО_2_-NP + SiО_2_-NP	0.4391 + 0.0061 *^, +, х^
Al_2_О_3_-NP + TiО_2_-NP + SiО_2_-NP (half doses)	0.4849 + 0.0068 *^, +, х, @^
Al_2_О_3_-NP + TiО_2_-NP + SiО_2_-NP (full doses)	0.6430 + 0.0189 *^, +, х, @^
Al_2_О_3_-NP + TiО_2_-NP + SiО_2_-NP + BPC	0.4742 + 0.0067 *^, +, х, @^
BPC	0.4143 + 0.0047
Control	0.4023 + 0.0064

The signs denote values differing statistically significantly (*p* < 0.05 by ANOVA test) as follows: * from the control value, ^+^ from the value under exposure to TiО_2_-NP, ^х^ from the value under exposure to SiО_2_-NP, ^@^ from the value under exposure to Аl_2_О_3_-NP. Also, there is a statistically significant difference between the groups administered the ternary MеO-NP combination in full and half doses, and in full dose and in the same dose with background administration of the BPC.

**Table 13:** Doses and Mode of Administration of the Bioprotectors Tested in Our Experiment.

Bioprotectors	Estimated Dosage and Mode of Administration
Apple pectin	1 g/kg (added to the fodder)
Sodium glutamate	160 mg per rat (as a 1.5% drink instead of water)
Glycine	12 mg per rat (added to the food)
*N*-Acetylcysteine	30 mg per rat (added to the food)
Vitamin C	4.4 mg per rat (added to the food)
Vitamin E	0.84 mg per rat (added to the food)
Selenium	4.0 mcg per rat (added to the food)
Commercial fish oil rich in vitamin A and omega 3 PUFA	1 drop per rat (sublingually)
Potassium iodide	4.0 mcg per rat (added to the food)
Calcium carbonate	160 mg per rat (added to the food)

## Supplementary Materials

Supplementary materials can be found at http://www.mdpi.com/1422-0067/19/3/837/s1.
