# Supplementary material for: Combined Subchronic Toxicity of Aluminum (III), Titanium (IV) and Silicon (IV) Oxide Nanoparticles and Its Alleviation with a Complex of Bioprotectors
Source: Int J Mol Sci. 2018 Mar 13;19(3):837. doi: 10.3390/ijms19030837 (PMC5877698; doi:10.3390/ijms19030837)
Supplement: Supplementary file 1 [file ijms-19-00837-s001.docx]

Supplementary Materials

In this document the we present some factual results of the experiment (Tables S1 and S2) and of their mathematical analysis (Figures S1-S6) which are not included in the body of the paper **“**Combined Subchronic Toxicity of Aluminum (III), Titanium (IV) and Silicon (IV) Oxide Nanoparticles and Its Alleviation with a Complex of Bioprotectors” (by Ilzira A. Minigalieva et al.) for the sake of making it more concise but can give additional foundation for, and illustration of the postulates discussed in that paper. Therefore here we refrain from repeating the same discussion but still explain inferences from each isobologram in Figires’ legends.

**Table S1.** Whole entity of measured Functional and Biochemical Indices of Rat Organism Status after 18 (during 6 Weeks) Intraperitoneal Injections of Suspensions of Various МеO-NP Species Administered Individually or in Binary Combinations (x ± s.e.).

| Index | Control | Al_2_O_3_ | TiO_2_ | SiO_2_ | Al_2_O_3_+  TiO_2_ | Al_2_O_3_+  SiO_2_ | TiO_2_+  SiO_2_ |
| --- | --- | --- | --- | --- | --- | --- | --- |
|  |  | Group 1 | Group 2 | Group 3 | Group 4 | Group 5 | Group 6 |
| Initial body mass, g | 292.27 ±  5.02 | 289.55 ±  7.93 | 287.50 ±  7.40 | 285.91 ±  9.17 | 287.08 ±  7.29 | 290.83 ±  7.53 | 291.67 ±  7.47 |
| Final body mass, g | 332.27 ±  7.93 | 333.18 ±  7.70 | 330.46 ±  9.33 | 322.27 ±  8.70 | 320.00 ±  3.59 | 322.50 ±  5.28 | 324.17 ±  7.76 |
| Body mass gain, % | 15.13 ±  1.85 | 17.30 ±  2.74 | 15.66 ±  1.69 | 13.14 ±  2.51 | 13.41 ±  2.07 | 14.70 ±  1.86 | 12.58 ±  2.09 |
| Number of head-dips into holes during 3 min | 4.73 ±  0.94 | 2.64 ±  0.64***** | 1.92 ±  0.36***** | 2.82 ±  0.64 | 5.08 ±  1.02**^2^** | 3.00 ±  0.59 | 4.42 ±  0.67**^2^** |
| Number of squares crossed during 3 min | 8.18 ±  1.25 | 5.82 ±  1.09 | 5.67 ±  0.99 | 7.00 ±  0.86 | 7.58 ±  1.17 | 5.00 ±  0.55***** | 7.58 ±  1.19 |
| Temporal summation of sub-threshold impulses, sec, | 14.27 ±  1.29 | 13.42 ±  1.19 | 14.34 ±  0.99 | 12.45 ±  0.91 | 15.35 ±  0.85 | 14.88 ±  1.08 | 13.39 ±  0.86 |
| Left kidney mass, g/100 g body mass | 0.30 ±  0.01 | 0.28 ±  0.01 | 0.30 ±  0.01**^1^** | 0.30 ±  0.01**^1^** | 0.30 ±  0.01**^1^** | 0.29 ±  0.01 | 0.30 ±  0.01 |
| Right kidney mass, g/100 g body mass | 0.31 ±  0.01 | 0.28 ±  0.01***** | 0.30 ±  0.01**^1^** | 0.30 ±  0.01**^1^** | 0.30 ±  0.01**^1^** | 0.30 ±  0.01**^1^** | 0.30 ±  0.01 |
| Liver mass, g/100 g body mass | 3.08 ±  0.13 | 3.14 ±  0.16 | 3.08 ±  0.12 | 3.08 ±  0.10 | 3.22 ±  0.18 | 3.22 ±  0.16 | 3.19 ±  0.13 |
| Spleen mass, g/100 g body mass | 0.17 ±  0.01 | 0.20 ±  0.01 | 0.17 ±  0.01**^1^** | 0.19 ±  0.01 | 0.18 ±  0.01 | 0.17 ±  0.01**^13^** | 0.18 ±  0.01 |
| Left testicle mass, g/100 g body mass | 0.55 ±  0.02 | 0.52 ±  0.01 | 0.53 ±  0.01 | 0.55 ±  0.01 | 0.55 ±  0.01 | 0.55 ±  0.01 | 0.53 ±  0.01 |
| Right testicle mass, g/100 g body mass | 0.55 ±  0.02 | 0.51 ±  0.01 | 0.52 ±  0.02 | 0.55 ±  0.01**^1^** | 0.54 ±  0.01 | 0.55 ±  0.01**^1^** | 0.53 ±  0.01 |
| Brain mass, g/100 g body mass | 0.62 ±  0.01 | 0.59 ±  0.01***** | 0.58 ±  0.02***** | 0.63 ±  0.01**^12^** | 0.61 ±  0.01 | 0.61 ±  0.01 | 0.61 ±  0.01 |
| Hemoglobin, g/L | 158.89 ±  1.16 | 141.14 ±  1.99***** | 149.00 ±  3.64**^1^*** | 149.71 ±  2.74**^1^*** | 147.33 ±  2.87***** | 146.00 ±  1.51***** | 151.25 ±  2.45***** |
| Erythrocytes, 10^12^ cells/L | 7.93 ±  0.16 | 7.68 ±  0.32 | 7.23 ±  0.13***** | 7.58 ±  0.14 | 7.61 ±  0.24 | 7.48 ±  0.11***** | 7.52 ±  0.10***** |
| Average erythrocyte volume, µm^3^ | 54.69 ±  0.86 | 55.05 ±  0.87 | 54.29 ±  0.85 | 54.34 ±  0.51 | 52.17 ±  0.67**^12^*** | 52.36 ±  0.57**^13^*** | 54.94 ±  0.34 |
| Reticulocytes, ‰ | 13.63 ±  1.65 | 25.64 ±  2.32***** | 32.60 ±  3.01***** | 26.63 ±  1.66***** | 29.90 ±  1.28***** | 25.86 ±  1.61***** | 31.67 ±  2.73***** |
| Hematocrit, % | 21.54 ±  0.21 | 19.73 ±  0.26***** | 20.21 ±  0.52***** | 20.60 ±  0.40***** | 20.03 ±  0.69***** | 19.59 ±  0.26***** | 20.65 ±  0.28***** |
| Thrombocytes,  10^3^/µL | 847.25 ±  25.41 | 860.00 ±  48.20 | 910.25 ±  67.20 | 857.25 ±  33.92 | 831.75 ±  54.09 | 926.57 ±  27.89 | 880.50 ±  34.53 |
| Thrombocrit, % | 0.23 ±  0.02 | 0.26 ±  0.02 | 0.28 ±  0.02 | 0.26 ±  0.01 | 0.24 ±  0.02 | 0.27 ±  0.01 | 0.25 ±  0.01 |
| Leukocytes, 10^3^/µL | 7.20 ±  0.37 | 8.98 ±  0.86***** | 8.40 ±  0.43***** | 7.69 ±  0.67 | 9.10 ±  1.03 | 9.40 ±  0.83***** | 7.85 ±  0.67 |
| Eosinophils,% | 2.20 ±  0.29 | 2.88 ±  0.61 | 2.38 ±  0.38 | 3.25 ±  0.73 | 3.13 ±  0.48 | 3.57 ±  0.87 | 2.13 ±  0.40 |
| Segmented neutrophils, % | 19.50 ±  0.64 | 19.88 ±  1.38 | 20.63 ±  1.15 | 18.88 ±  1.51 | 20.00 ±  0.82 | 20.43 ±  1.91 | 20.50 ±  0.98 |
| Banded neutrophils, % | 1.50 ±  0.17 | 1.88 ±  0.30 | 0.88 ±  0.13**^1^*** | 1.88 ±  0.40**^2^** | 1.00 ±  0.00**^1^*** | 2.29 ±  0.29***** | 1.38 ±  0.18^2^ |
| Monocytes,% | 6.20 ±  0.39 | 6.13 ±  0.61 | 6.00 ±  0.53 | 6.75 ±  0.49 | 6.75 ±  0.49 | 6.86 ±  0.34 | 6.38 ±  0.42 |
| Lymphocytes,% | 70.60 ±  0.95 | 69.25 ±  1.91 | 70.13 ±  1.38 | 69.25 ±  2.27 | 69.13 ±  0.91 | 66.71 ±  2.60 | 69.63 ±  1.13 |
| Succinate dehydrogenase (SDH) in blood lymphocytes, number of formazan granules per 50 cells | 589.45  ±  16.55 | 536.73  ±  17.43***** | 539.36  ±  16.94***** | 553.55  ±  17.46 | 562.67  ±  15.74 | 551.55  ±  20.54 | 530.42  ±  16.03***** |
| γ-glutamyl transpeptidase (GGTP), IU/L | 2.26 ±  0.69 | 1.14 ±  0.38 | 1.14 ±  0.40 | 1.86 ±  0.69 | 2.48 ±  0.45**^12^** | 3.98 ±  0.99**^1^** | 0.74 ±  0.30 |
| Glucose, mol/L | 7.09 ±  0.26 | 6.80 ±  0.21 | 6.33 ±  0.25***** | 6.80 ±  0.30 | 6.10 ±  0.30***** | 6.64 ±  0.18 | 7.04 ±  0.29 |
| Ceruloplasmin in blood serum, mg per 100 mL | 33.14 ±  1.13 | 38.09 ±  1.56***** | 42.03 ±  2.05***** | 40.39 ±  1.50***** | 44.06 ±  1.53**^1^*** | 46.22 ±  2.35**^13^*** | 42.88 ±  1.44***** |
| Malonyl dialdehyde (MDA) in blood serum, µmol/L | 3.51 ±  0.49 | 3.99 ±  0.19 | 3.16 ±  0.28**^1^** | 3.37 ±  0.31 | 3.56 ±  0.48 | 5.10 ±  0.37**^13^*** | 4.57 ±  0.19**^23^*** |
| Catalase in blood serum, µmol/L | 1.34 ±  0.25 | 1.30 ±  0.22 | 1.20 ±  0.27 | 1.12 ±  0.22 | 1.31 ±  0.22 | 1.18 ±  0.24 | 0.65 ±  0.12***** |
| Reduced glutathione in whole blood, µmol/L | 26.82 ±  1.19 | 27.75 ±  1.36 | 26.45 ±  1.18 | 22.89 ±  1.841 | 26.20 ±  0.87 | 28.44 ±  1.473 | 26.00 ±  1.39 |
| SH-groups in blood plasma, mmol/L | 37.33 ±  7.38 | 37.23 ±  5.67 | 40.32 ±  6.88 | 34.81 ±  5.11 | 36.93 ±  6.04 | 43.21 ±  6.99 | 42.72 ±  6.90 |
| Total protein content of blood serum, g/L | 80.47 ±  1.42 | 76.81 ±  1.97 | 75.43 ±  1.40***** | 75.36 ±  2.00***** | 80.49 ±  2.01 | 78.20 ±  1.33 | 78.93 ±  2.15 |
| Albumin content of blood serum, g/L | 44.34 ±  0.61 | 39.49 ±  0.81***** | 40.28 ±  1.35***** | 40.25 ±  1.44***** | 41.31 ±  1.05***** | 39.58 ±  0.67***** | 40.18 ±  1.24***** |
| Globulins of blood serum, g/L | 36.13 ±  1.22 | 37.33 ±  2.07 | 35.15 ±  1.65 | 35.49 ±  1.76 | 39.18 ±  1.54 | 38.63 ±  1.06 | 38.75 ±  1.53 |
| A/G index | 1.24 ±  0.04 | 1.08 ±  0.06***** | 1.17 ±  0.07 | 1.14 ±  0.06 | 1.06 ±  0.05***** | 1.03 ±  0.03***** | 1.05 ±  0.05***** |
| AST activity in blood serum, IU/L | 218.44 ±  17.65 | 257.93 ±  12.42 | 261.38 ±  26.36 | 193.10 ±  22.6112 | 264.61 ±  25.72 | 236.81 ±  17.78 | 187.39 ±  5.072 |
| ALT activity in blood serum, IU/L | 70.82 ±  3.24 | 72.70 ±  3.10 | 69.00 ±  4.19 | 58.55 ±  4.281***** | 66.46 ±  4.41 | 66.50 ±  1.66 | 63.94 ±  3.32 |
| De Ritis coefficient | 3.12 ±  0.24 | 3.57 ±  0.18 | 3.75 ±  0.23 | 3.31 ±  0.31 | 3.76 ±  0.39 | 3.55 ±  0.23 | 3.01 ±  0.23 |
| Alkaline phosphatase, IU/L | 193.64 ±  13.08 | 215.71 ±  14.74 | 216.61 ±  23.36 | 212.59 ±  26.36 | 222.55 ±  13.71 | 240.48 ±  21.89***** | 236.53 ±  10.62***** |
| Creatinine in blood serum, µmol/L | 36.33 ±  1.46 | 33.64 ±  1.09 | 30.80 ±  0.711***** | 32.40 ±  1.29***** | 34.46 ±  1.71 | 34.50 ±  1.49 | 33.89 ±  1.352 |
| Bilirubin in blood serum, μmol/L | 1.14 ±  0.13 | 0.90 ±  0.15 | 1.05 ±  0.15 | 1.00 ±  0.10 | 1.31 ±  0.13 | 1.09 ±  0.14 | 0.98 ±  0.14 |
| Concentration of Ca^2+^ in blood serum, mol/L | 2.61 ±  0.03 | 2.53 ±  0.02***** | 2.54 ±  0.05 | 2.55 ±  0.04 | 2.56 ±  0.04 | 2.57 ±  0.03 | 2.52 ±  0.05 |
| Follicle stimulating hormone in blood serum, IU/L | 0.14 ±  0.02 | 0.11 ±  0.00 | 0.11 ±  0.01 | 0.12 ±  0.01 | 0.13  ± 0.01 | 0.11  ± 0.01 | 0.11  ± 0.01 |
| Luteinizing hormone in blood serum, IU/L | 0.13 ±  0.02 | 0.14 ±  0.02 | 0.24 ±  0.10 | 1.31 ±  0.94 | 0.94 ±  0.58 | 0.35 ±  0.21 | 0.77 ±  0.63 |
| Prolactin in blood serum, IU / L | 7.85 ±  0.62 | 6.38 ±  0.58 | 10.22 ±  2.59 | 9.90 ±  1.91 | 15.71 ±  4.51 | 8.22 ±  0.82 | 14.73 ±  5.25 |
| Testosterone in blood serum, nmol/L | 8.20 ±  3.40 | 12.50 ±  3.21 | 26.51 ±  9.95 | 13.26 ±  5.19 | 9.77 ±  2.10 | 8.45 ±  4.01 | 16.28 ± 4.27 |
| Lactate dehydrogenase (LDH) in blood serum, IU/L | 1904.10 ±  296.03 | 2402.25 ±  277.51 | 2924.38 ±  530.17 | 1781.00 ±  349.35 | 2208.25 ±  290.47 | 2119.50 ±  305.84 | 1628.50  ±  149.27 |
| Uric acid in blood serum, µmol/L | 120.50 ±  10.86 | 154.88 ±  13.53 | 135.50 ±  15.29 | 128.25 ±  17.67 | 121.00 ±  11.99 | 122.00 ±  9.19 | 115.75 ±  8.69 |
| Urea in blood serum, mmol/L | 4.44 ±  0.34 | 3.69 ±  0.28 | 3.89 ±  0.45 | 3.29 ±  0.40***** | 3.59 ±  0.36 | 3.71 ±  0.27 | 3.73 ±  0.37 |
| Daily diuresis, mL | 29.67 ±  4.36 | 21.17 ±  2.39 | 26.43 ±  3.88 | 28.43 ±  5.73 | 33.00 ±  2.50**^1^** | 24.86 ±  2.20 | 31.71 ±  5.64 |
| Coproporphyrin in urine, nmol/L | 162.42 ±  31.78 | 135.77 ±  21.32 | 120.40 ±  40.13 | 185.31 ±  74.84 | 135.37 ±  62.57 | 111.83 ±  39.84 | 155.14 ±  31.38 |
| Daily coproporphyrin in urine, µmol | 8.14 ±  3.60 | 6.47 ±  1.38 | 5.87 ±  3.10 | 3.73 ±  0.67 | 4.63 ±  2.23 | 5.42 ±  2.46 | 5.91 ±  2.02 |
| δ–ALA in urine, µg/mL | 14.11 ±  3.52 | 15.63  ± 5.08 | 11.99  ± 4.40 | 10.90  ± 4.47 | 13.87  ± 4.37 | 9.90  ± 3.98 | 12.61  ± 2.99 |
| Creatinine in urine, mmol/L | 1.57 ±  0.11 | 2.56 ±  0.27***** | 2.15 ±  0.35 | 1.91 ±  0.161 | 1.54 ±  0.141 | 1.92 ±  0.131 | 1.85 ±  0.17 |
| Endogenous creatinine clearance | 1.40 ±  0.15 | 1.58 ±  0.18 | 1.90 ±  0.24 | 1.74 ±  0.19 | 1.50 ±  0.23 | 1.38 ±  0.08 | 1.60 ±  0.19 |
| Protein in urine, g/L | 190.43 ±  29.63 | 298.45 ±  32.35***** | 216.55 ±  33.41 | 180.93 ±  17.09**^1^** | 196.13 ±  20.43**^1^** | 193.36 ±  20.67**^1^** | 211.45 ±  37.18 |
| Urine pH | 7.17 ±  0.17 | 6.50 ±  0.26***** | 7.33 ±  0.40 | 7.36 ±  0.30 | 6.79 ±  0.15 | 7.00 ±  0.29 | 6.93 ±  0.17 |
| Urea in urine, mmol/L | 229.30 ±  16.00 | 319.41 ±  29.85***** | 278.06 ±  46.97 | 240.15 ±  25.77**^1^** | 211.22 ±  17.47**^1^** | 262.08 ±  19.23 | 238.15 ±  24.71 |
| Uric acid in urine, µmol/L | 234.00 ±  38.97 | 319.50 ±  45.51 | 292.50 ±  105.70 | 304.71 ±  93.85 | 204.29 ±  78.30 | 201.86 ±  64.52 | 213.00 ±  51.37 |

*Note: The asterisk* ***** *designates the values which are statistically significantly different from the respective control ones, and the superscript numbers those statistically significantly different from the corresponding groups denoted with a corresponding number (p<0.05 by ANOVA test)*

**Table S2.** Whole entity of measured Functional and Biochemical Indices of Rat Organism Status after 18 (during 6 weeks) Repeated Intraperitoneal Injections of Suspensions of Various МеО-NP Species Administered in Binary and Ternary Combinations (x ± s.e.).

| Index | Control | Al_2_O_3_+  TiO_2_ | Al_2_O_3_+  SiO_2_ | TiO_2_+  SiO_2_ | Al_2_O_3_+  SiO_2_+ TiO_2_ | Al_2_O_3_+  SiO_2_+ TiO_2_ and BPC | BPC |
| --- | --- | --- | --- | --- | --- | --- | --- |
|  |  | Group 1 | Group 2 | Group 3 | Group 4 | Group 5 | Group 6 |
| Initial body mass, g | 292.27 ±  5.02 | 287.08 ±  7.29 | 290.83 ±  7.53 | 291.67 ±  7.47 | 290.00 ±  5.56 | 295.00 ±  5.16 | 295.00 ±  7.03 |
| Final body mass, g | 332.27 ±  7.93 | 320.00 ±  3.59 | 322.50 ±  5.28 | 324.17 ±  7.76 | 334.55 ±  8.08 | 346.50 ±  4.72 | 354.50 ±  7.40 |
| Body mass gain, % | 15.13 ±  1.85 | 13.41 ±  2.07 | 14.70 ±  1.86 | 12.58 ±  2.09 | 15.40 ±  2.02 | 18.34 ±  2.00 | 20.32 ±  1.41* |
| Number of head-dips into holes during 3 min | 4.73 ±  0.94 | 5.08 ±  1.02 | 3.00 ±  0.59 | 4.42 ±  0.67 | 3.27 ±  0.78 | 3.80 ±  0.98 | 4.40 ±  0.76 |
| Number of squares crossed during 3 min | 8.18 ±  1.25 | 7.58 ±  1.17 | 5.00 ±  0.55* | 7.58 ±  1.19 | 6.50 ±  1.09 | 4.90 ±  1.14 | 7.78 ±  1.16 |
| Temporal summation of sub-threshold impulses, sec, | 14.27 ±  1.29 | 15.35 ±  0.85 | 14.88 ±  1.08 | 13.39 ±  0.86 | 14.41 ±  0.97 | 13.47 ±  0.88 | 11.41 ±  1.18 |
| Left kidney mass, g/100 g body mass | 0.30 ±  0.01 | 0.30 ±  0.01 | 0.29 ±  0.01 | 0.30 ±  0.01 | 0.28 ±  0.01 | 0.30 ±  0.01 | 0.30 ±  0.01 |
| Right kidney mass, g/100 g body mass | 0.31 ±  0.01 | 0.30 ±  0.01 | 0.30 ±  0.01 | 0.30 ±  0.01 | 0.29 ±  0.01 | 0.30 ±  0.01 | 0.30 ±  0.01 |
| Liver mass, g/100 g body mass | 3.08 ±  0.13 | 3.22 ±  0.18 | 3.22 ±  0.16 | 3.19 ±  0.13 | 3.09 ±  0.16 | 3.04 ±  0.09 | 3.21 ±  0.16 |
| Spleen mass, g/100 g body mass | 0.17 ±  0.01 | 0.18 ±  0.01 | 0.17 ±  0.01 | 0.18 ±  0.01 | 0.18 ±  0.01 | 0.19 ±  0.00 | 0.18 ±  0.01 |
| Left testicle mass, g/100 g body mass | 0.55 ±  0.02 | 0.55 ±  0.01 | 0.55 ±  0.01 | 0.53 ±  0.01 | 0.53 ±  0.02 | 0.55 ±  0.02 | 0.52 ±  0.01 |
| Right testicle mass, g/100 g body mass | 0.55 ±  0.02 | 0.54 ±  0.01 | 0.55 ±  0.01 | 0.53 ±  0.01 | 0.53 ±  0.02 | 0.55 ±  0.02 | 0.52 ±  0.01 |
| Brain mass, g/100 g body mass | 0.62 ±  0.01 | 0.61 ±  0.01 | 0.61 ±  0.01 | 0.61 ±  0.01 | 0.59 ±  0.01* | 0.59 ±  0.01* | 0.61 ±  0.01 |
| Hemoglobin, g/L | 158.89 ±  1.16 | 147.33 ±  2.87* | 146.00 ±  1.51* | 151.25 ±  2.45* | 147.75 ±  2.28* | 143.26 ±  1.49* | 151.14 ±  1.92* |
| Erythrocytes, 10^12^ cells/L | 7.93 ±  0.16 | 7.61 ±  0.24 | 7.48 ±  0.11* | 7.52 ±  0.10* | 7.83 ±  0.17 | 7.75 ±  0.14 | 7.42 ±  0.10* |
| Average erythrocyte volume, µm^3^ | 54.69 ±  0.86 | 52.17 ±  0.67* | 52.36 ±  0.57* | 54.94 ±  0.34 | 51.73 ±  0.93* | 51.88 ±  0.82* | 56.60 ±  0.61 |
| Reticulocytes, ‰ | 13.63 ±  1.65 | 29.90 ±  1.28* | 25.86 ±  1.61* | 31.67 ±  2.73* | no data | 26.00 ±  0.88* | 17.67 ±  4.78 |
| Hematocrit, % | 21.54 ±  0.21 | 20.03 ±  0.69* | 19.59 ±  0.26* | 20.65 ±  0.28* | 20.20 ±  0.30* | 20.09 ±  0.45* | 21.00 ±  0.36 |
| Thrombocytes,10^3^/µL | 847.25 ±  25.41 | 831.75 ±  54.09 | 926.57 ±  27.89 | 880.50 ±  34.53 | 882.25 ±  36.87 | 979.56 ±  26.63*^4^ | 890.25 ±  36.39 |
| Thrombocrit, % | 0.23 ±  0.02 | 0.24 ±  0.02 | 0.27 ±  0.01 | 0.25 ±  0.01 | 0.26 ±  0.01 | 0.29 ±  0.01*^4^ | 0.27 ±  0.01 |
| Leukocytes, 10^3^/µL | 7.20 ±  0.37 | 9.10 ±  1.03 | 9.40 ±  0.83* | 7.85 ±  0.67 | 7.78 ±  0.66 | 10.04 ±  0.93*^4^ | 7.88 ±  0.46 |
| Eosinophils,% | 2.20 ±  0.29 | 3.13 ±  0.48 | 3.57 ±  0.87 | 2.13 ±  0.40 | 3.00 ±  0.42 | 3.22 ±  0.36* | 3.13 ±  0.69 |
| Segmented neutrophils, % | 19.50 ±  0.64 | 20.00 ±  0.82 | 20.43 ±  1.91 | 20.50 ±  0.98 | 20.75 ±  1.05 | 20.78 ±  1.04 | 18.88 ±  1.08 |
| Banded neutrophils, % | 1.50 ±  0.17 | 1.00 ±  0.00* | 2.29 ±  0.29* | 1.38 ±  0.18 | 1.63 ±  0.26 | 1.67 ±  0.33 | 1.25 ±  0.16 |
| Monocytes,% | 6.20 ±  0.39 | 6.75 ±  0.49 | 6.86 ±  0.34 | 6.38 ±  0.42 | 6.25 ±  0.45 | 6.44 ±  0.50 | 6.00 ±  0.42 |
| Lymphocytes,% | 70.60 ±  0.95 | 69.13 ±  0.91 | 66.71 ±  2.60 | 69.63 ±  1.13 | 68.50 ±  1.24 | 67.89 ±  0.98 | 70.75 ±  1.31 |
| Succinate dehydrogenase (SDH) in blood lymphocytes, number of formazan granules per 50 cells | 589.45  ±  16.55 | 562.67  ±  15.74 | 551.55  ±  20.54 | 530.42  ±  16.03* | 561.64  ±  15.99 | 559.50  ±  16.67 | 578.90  ±  14.48 |
| γ-glutamyl transpeptidase (GGTP), IU/L | 2.26 ±  0.69 | 2.48 ±  0.45 | 3.98 ±  0.99 | 0.74 ±  0.30 | 1.10 ±  0.66 | 1.23 ±  0.40 | 1.41 ±  0.69 |
| Glucose, mol/L | 7.09 ±  0.26 | 6.10 ±  0.30* | 6.64 ±  0.18 | 7.04 ±  0.29 | 7.08 ±  0.18 | 7.13 ±  0.35 | 7.04 ±  0.17 |
| Ceruloplasmin in blood serum, mg per 100 mL | 33.14 ±  1.13 | 44.06 ±  1.53* | 46.22 ±  2.35* | 42.88 ±  1.44* | 42.61 ±  1.88* | 38.36 ±  2.71 | 30.54 ±  1.82 |
| Malonyl dialdehyde (MDA) in blood serum, µmol/L | 3.51 ±  0.49 | 3.56 ±  0.48 | 5.10 ±  0.37* | 4.57 ±  0.19* | 4.28 ±  0.29 | 4.20 ±  0.28 | 4.09 ±  0.19 |
| Catalase in blood serum, µmol/L | 1.34 ±  0.25 | 1.31 ±  0.22 | 1.18 ±  0.24 | 0.65 ±  0.12* | 1.10 ±  0.21 | 0.74 ±  0.25 | 0.86 ±  0.31 |
| Reduced glutathione in whole blood, µmol/L | 26.82 ±  1.19 | 26.20 ±  0.87 | 28.44 ±  1.473 | 26.00 ±  1.39 | 22.55 ±  1.41* | 26.39 ±  1.36^4^ | 28.17 ±  1.35 |
| SH-groups in blood plasma, mmol/L | 37.33 ±  7.38 | 36.93 ±  6.04 | 43.21 ±  6.99 | 42.72 ±  6.90 | 40.20 ±  6.66 | 37.34 ±  7.64 | 34.13 ±  6.64 |
| Total protein content of blood serum, g/L | 80.47 ±  1.42 | 80.49 ±  2.01 | 78.20 ±  1.33 | 78.93 ±  2.15 | 79.91 ±  1.82 | 81.00 ±  1.79 | 80.46 ±  1.59 |
| Albumin content of blood serum, g/L | 44.34 ±  0.61 | 41.31 ±  1.05* | 39.58 ±  0.67* | 40.18 ±  1.24* | 41.91 ±  0.88* | 43.38 ±  0.94 | 44.91 ±  0.90 |
| Globulins of blood serum, g/L | 36.13 ±  1.22 | 39.18 ±  1.54 | 38.63 ±  1.06 | 38.75 ±  1.53 | 38.00 ±  1.40 | 37.62 ±  1.45 | 35.55 ±  1.30 |
| A/G index | 1.24 ±  0.04 | 1.06 ±  0.05* | 1.03 ±  0.03* | 1.05 ±  0.05* | 1.11 ±  0.04* | 1.17 ±  0.05 | 1.27 ±  0.05 |
| AST activity in blood serum, IU/L | 218.44 ±  17.65 | 264.61 ±  25.72 | 236.81 ±  17.78 | 187.39 ±  5.072 | 213.91 ±  17.83 | 214.32 ±  18.45 | 205.88 ±  10.52 |
| ALT activity in blood serum, IU/L | 70.82 ±  3.24 | 66.46 ±  4.41 | 66.50 ±  1.66 | 63.94 ±  3.32 | 66.75 ±  3.55 | 83.09 ±  5.13*^4^ | 84.98 ±  4.69* |
| De Ritis coefficient | 3.12 ±  0.24 | 3.76 ±  0.39 | 3.55 ±  0.23 | 3.01 ±  0.23 | 3.22 ±  0.25 | 2.67 ±  0.31 | 2.50 ±  0.24 |
| Alkaline phosphatase, IU/L | 193.64 ±  13.08 | 222.55 ±  13.71 | 240.48 ±  21.89* | 236.53 ±  10.62* | 200.30 ±  12.15 | 209.78 ±  21.48 | 261.99 ±  24.46* |
| Creatinine in blood serum, µmol/L | 36.33 ±  1.46 | 34.46 ±  1.71 | 34.50 ±  1.49 | 33.89 ±  1.352 | 35.39 ±  1.03 | 34.39 ±  1.25 | 34.54 ±  0.94 |
| Bilirubin in blood serum, μmol/L | 1.14 ±  0.13 | 1.31 ±  0.13 | 1.09 ±  0.14 | 0.98 ±  0.14 | 1.10 ±  0.16 | 1.06 ±  0.08 | 0.99 ±  0.17 |
| Concentration of Ca^2+^ in blood serum, mol/L | 2.61 ±  0.03 | 2.56 ±  0.04 | 2.57 ±  0.03 | 2.52 ±  0.05 | 2.58 ±  0.02 | 2.66 ±  0.06 | 2.68 ±  0.05 |
| Follicle stimulating hormone in blood serum, IU/L | 0,14 ±  0,02 | 0,13 ±  0,01 | 0,11 ±  0,01 | 0,11 ±  0,01 | 0.14 ±  0.01 | 0.14 ±  0.01 | 0.14 ±  0.01 |
| Luteinizing hormone in blood serum, IU/L | 0.13 ±  0.02 | 0.94 ±  0.58 | 0.35 ±  0.21 | 0.77 ±  0.63 | 0.12 ±  0.01 | 0.16 ±  0.02 | 0.15 ±  0.01 |
| Prolactin in blood serum, IU / L | 7,85 ±  0,62 | 15,71 ±  4,51 | 8,22  0,82 | 14,73 ±  5,25 | 6.84 ±  0.35 | 8.42 ±  0.86 | 7.52 ±  0.46 |
| Testosterone in blood serum, nmol/L | 8,20 ±  3,40 | 9,77 ±  2,10 | 8,45 ±  4,01 | 16,28 ±  4,27 | 13.34 ±  8.17 | 13.47 ±  7.99 | 8.41 ±  2.58 |
| Lactate dehydrogenase (LDH) in blood serum, IU/L | 1904.10 ±  296.03 | 2208.25 ±  290.47 | 2119.50 ±  305.84 | 1628.50 ±  149.27 | 1709.88 ±  246.58 | 1313.22 ±  259.49 | 1433.00 ±  179.78 |
| Uric acid in blood serum, µmol/L | 120.50 ±  10.86 | 121.00 ±  11.99 | 122.00 ±  9.19 | 115.75 ±  8.69 | 123.63 ±  9.61 | 128.89 ±  12.32 | 127.75 ±  11.33 |
| Urea in blood serum, mmol/L | 4.44 ±  0.34 | 3.59 ±  0.36 | 3.71 ±  0.27 | 3.73 ±  0.37 | 3.35 ±  0.42 | 4.93 ±  0.45^4^ | 4.49 ±  0.42 |
| Daily diuresis, mL | 29.67 ±  4.36 | 33.00 ±  2.50 | 24.86 ±  2.20 | 31.71 ±  5.64 | 26.86 ±  4.14 | 29.00 ±  6.52 | 32.40 ±  8.36 |
| Coproporphyrin in urine, nmol/L | 162.42 ±  31.78 | 135.37 ±  62.57 | 111.83 ±  39.84 | 155.14 ±  31.38 | 76.11 ±  24.08 | 176.13 ±  49.92 | 79.15 ±  26.76 |
| Daily coproporphyrin in urine, µmol | 8.14 ±  3.60 | 4.63 ±  2.23 | 5.42 ±  2.46 | 5.91 ±  2.02 | 4.34 ±  2.23 | 13.97 ±  6.94 | 2.76 ±  1.30 |
| δ–ALA in urine, µg/mL | 14.11 ±  3.52 | 13.87 ±  4.37 | 9.90 ±  3.98 | 12.61 ±  2.99 | 13.48 ±  5.22 | 13.21 ±  3.28 | 8.31 ±  1.84 |
| Creatinine in urine, mmol/L | 1.57 ±  0.11 | 1.54 ±  0.14 | 1.92 ±  0.13 | 1.85 ±  0.17 | 2.03 ±  0.19* | 1.74 ±  0.25 | 1.43 ±  0.21 |
| Endogenous creatinine clearance | 1.40 ±  0.15 | 1.50 ±  0.23 | 1.38 ±  0.08 | 1.60 ±  0.19 | 1.49 ±  0.19 | 1.46 ±  0.18 | 1.50 ±  0.13 |
| Protein in urine, g/L | 190.43 ±  29.63 | 196.13 ±  20.43 | 193.36 ±  20.67 | 211.45 ±  37.18 | 233.13 ±  30.83 | 354.33 ±  66.07 | 243.93 ±  22.83* |
| Urine pH | 7.17 ±  0.17 | 6.79 ±  0.15 | 7.00 ±  0.29 | 6.93 ±  0.17 | 6.93 ±  0.37 | 7.75 ±  0.25 | 7.500 ±  0.000 |
| Urea in urine, mmol/L | 229.30 ±  16.00 | 211.22 ±  17.47 | 262.08 ±  19.23 | 238.15 ±  24.71 | 289.74 ±  28.72 | 242.70 ±  39.98 | 222.15 ±  16.29 |
| Uric acid in urine, µmol/L | 234.00 ±  38.97 | 204.29 ±  78.30 | 201.86 ±  64.52 | 213.00 ±  51.37 | 216.00 ±  52.97 | 287.17 ±  109.69 | 209.00 ±  43.27 |

*Note: The asterisk* ***** *designates the values which are statistically significantly different from the control ones, and the superscript numbers mark the values which are statistically significantly different from the corresponding values of the groups denoted with a corresponding number (p<0.05 by ANOVA test)*


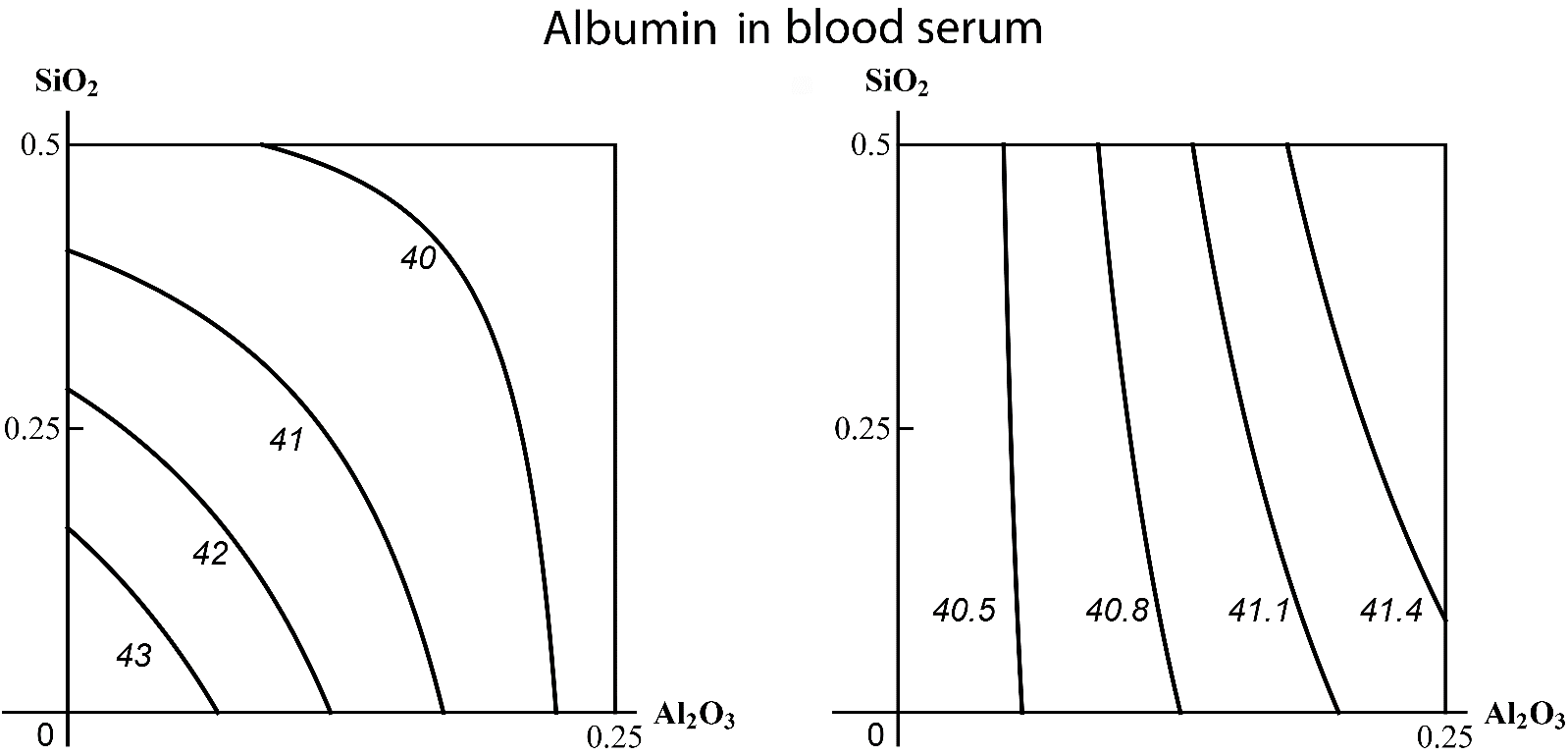


**Figure S1.** An example of three-factor toxicity falling into class “В”: (а) a unidirectional (subadditive) action of SiO2-NP + Al2O3-NP in the absence of a third factor on the albumin content of blood serum transforms into (b) a single-factor action of Al2O3-NP alone in the presence of simultaneously influencing TiO2-NPs. The numbers at the lines correspond to the magnitude of the effect (g/L).


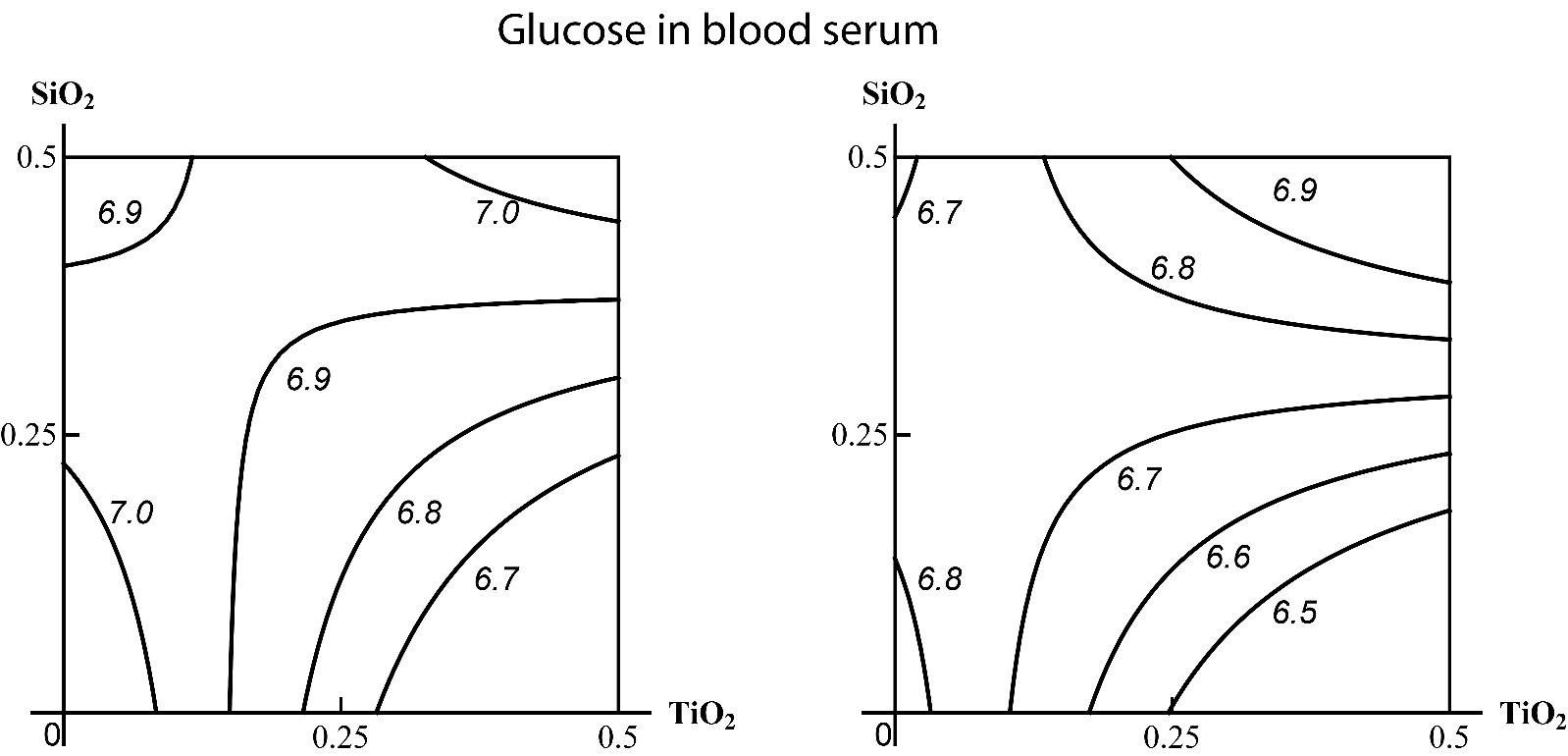


**Figure S2.** An example of three-factor toxicity falling into class “C”: the isoboles of combined action of SiO_2_-NP + TiO_2_-NP on the glucose content of blood serum virtually fully match (а) in the absence of a third factor and (b) in the presence of Al_2_O_3_-NP. The МеО-NP doses are plotted on the axes in mg per rat. The numbers at the lines correspond to the magnitude of the effect (µmol/L).


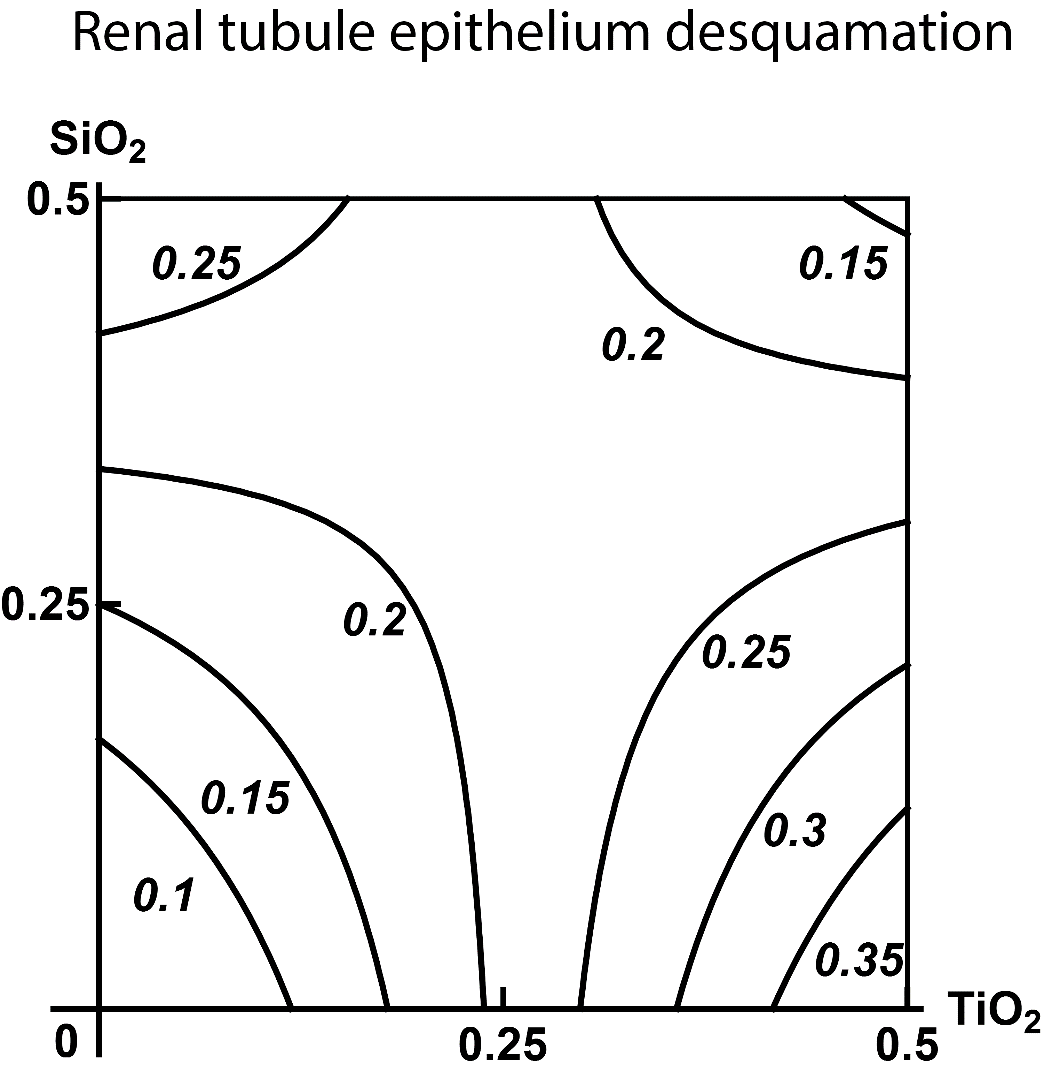


**Figure S3.** Isobologram of combined subchronic toxicity of SiO_2_-NP + TiO_2_-NP assessed by renal tubule epithelium desquamation: subadditivity of unidirectional action at lower doses of both MeО-NPs, superadditivity at maximum doses of bоth species, different variants of opposite action in combinations of low doses of one MeО-NP with high doses of the other. The axes represent doses of corresponding МеО-NPs in mg per rat; the numbers at the isoboles denote the magnitude of the effect (expressed in % - see the text).

| 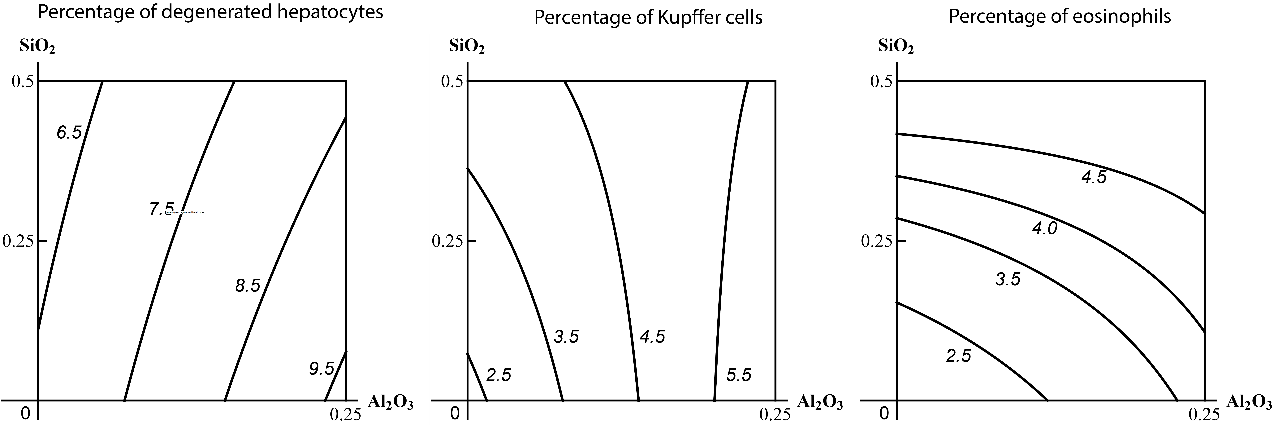 | | |
| --- | --- | --- |
| **a** | **b** | **c** |
| 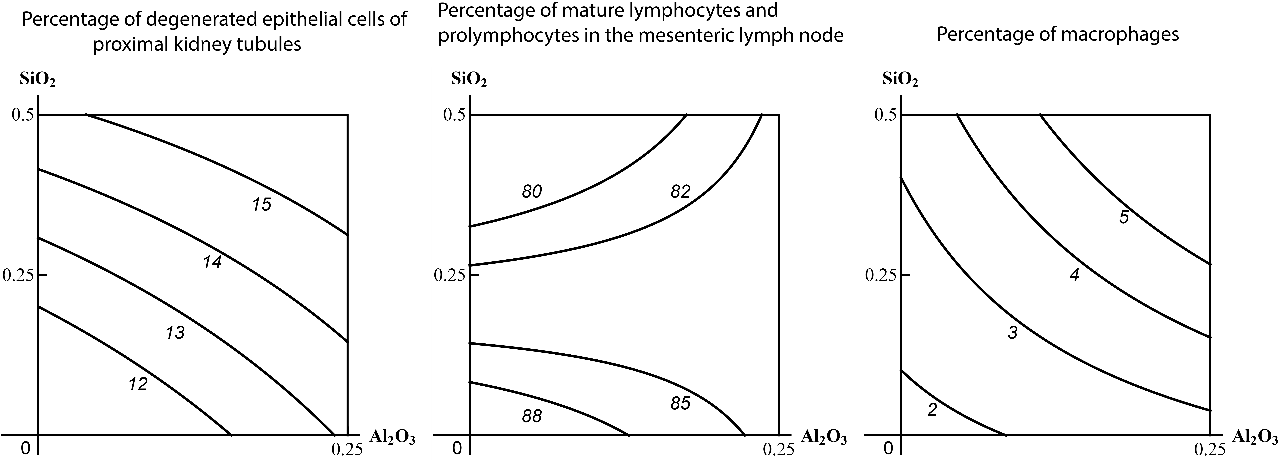 | | |
| **d** | **e** | **f** |

**Figure S4.** Examples of isobolograms of combined subchronic toxicity of Al_2_О_3_-NP+SiO_2_-NP assessed by: (а) impact on the percentage of degenerated hepatocytes in the liver tissue imprint (unidirectional action); (b) impact on the percentage of Kupffer cells in the same imprint (additivity of unidirectional action at low doses of Al_2_О_3_-NP transient to a single-factor action at higher doses); (c) impact on the percentage of eosinophils in the same imprint (subadditivity of unidirectional action); (d) impact on the percentage of degenerated epithelial cells of proximal kidney tubules in the kidney tissue imprint (additivity of unidirectional action); (e) impact on the percentage of mature lymphocytes and prolymphocytes in the mesenteric lymph node tissue imprint (subadditivity of unidirectional action at low doses of SiO_2_-NP transient to opposite action at higher doses); (е) impact on the percentage of macrophages in the same imprint (superadditivity of unidirectional action). The axes represent doses of SiO_2_-NP and Al_2_О_3_-NP in mg per rat; the numbers at the isoboles denote the magnitude of the effect (expressed in % - see the text).

| 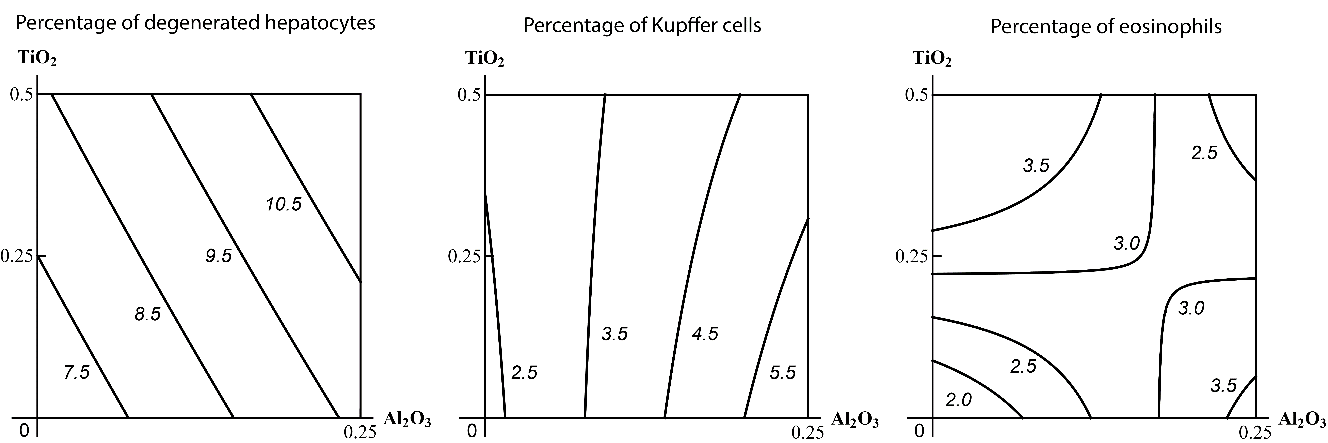 | | |
| --- | --- | --- |
| **a** | **b** | **c** |
| 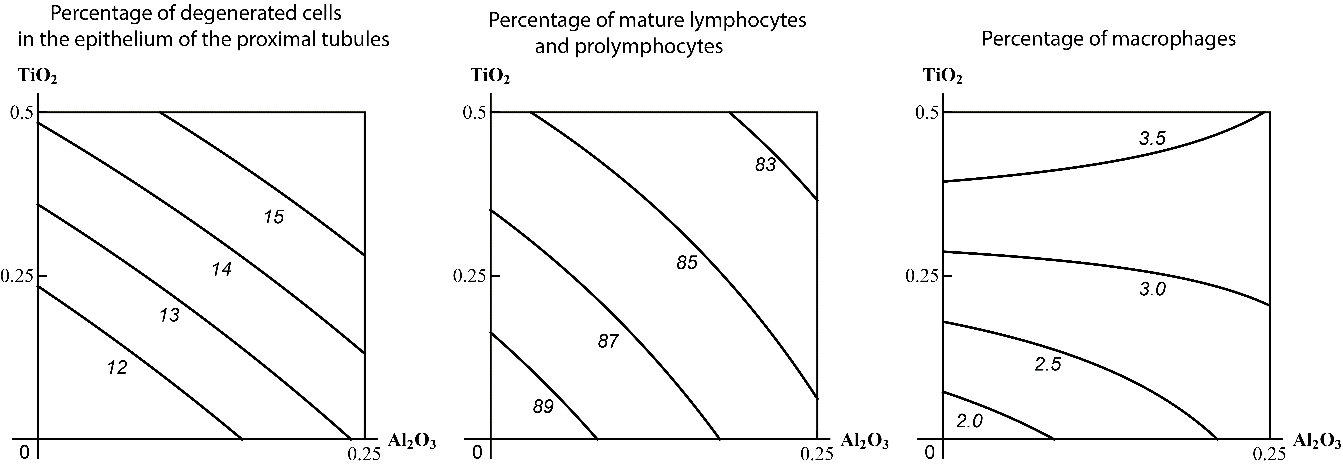 | | |
| **d** | **e** | **f** |

**Figure S5.** Examples of isoboles of combined subchronic toxicity of Al_2_О_3_-NP+ТiO_2_-NP assessed by: (а) impact on the percentage of degenerated hepatocytes in the liver tissue imprint (additivity of unidirectional action); (b) impact on the percentage of Kupffer cells in the same imprint (single-factor action); (c) impact on the percentage of eosinophils in the same imprint (subadditivity of unidirectional action at low doses and relatively high levels of effect; superadditivity at high doses and low levels of effect; opposite action at high doses of one of the toxicants and relatively high levels of effect);(d) impact on the percentage of degenerated cells in the epithelium of the proximal tubules in the kidney tissue imprint (additivity of unidirectional action); (e) impact on the percentage of mature lymphocytes and prolymphocytes in the mesenteric lymph node tissue imprint (additivity); (f) impact on the percentage of macrophages in the same imprint (largely additive unidirectional action with transformation into an opposite one). The axes represent corresponding doses of ТiO_2_-NP and Al_2_О_3_-NP in mg per rat; the numbers at the isobole denote the magnitude of the effect (expressed in % - see the text).


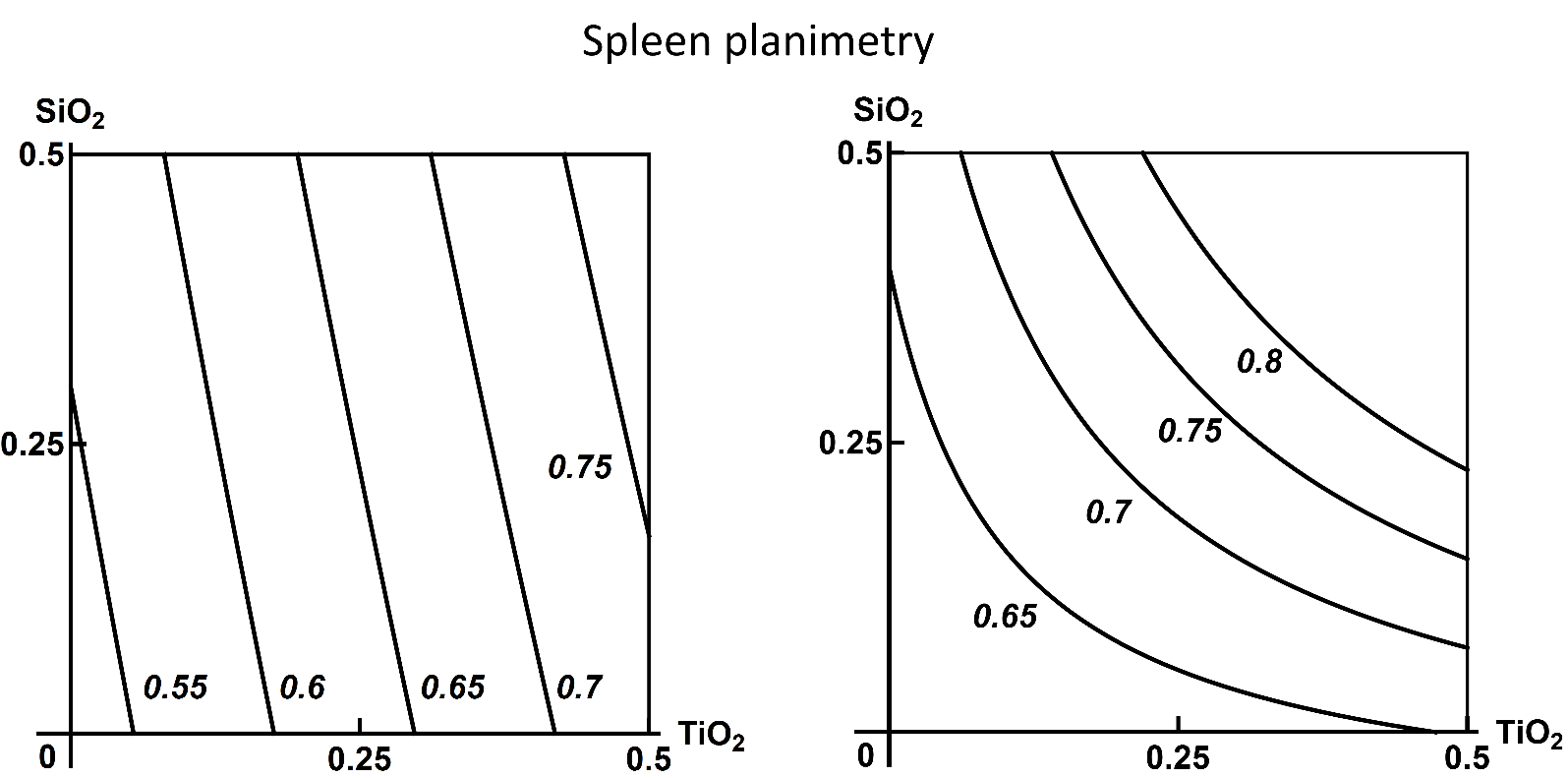


**Figure S6.**
